# The Prognostic Value of Pre-Procedural and Post-Procedural Inflammatory–Oxidative Stress Biomarkers in Acute Coronary Patients Undergoing Percutaneous Coronary Intervention: A Systematic Review and Meta-Analysis

**DOI:** 10.3390/ijms27083389

**Published:** 2026-04-09

**Authors:** Jonathan Samuel Matogu Tambunan, Citrawati Dyah Kencono Wungu, Hendri Susilo, Azizah Bonitha Zahrah Santoso, Anindita Azkia Fauzana, Pramudya Dhafa Hernandi, Albert Steven Purnama, Langgeng Agung Waskito, Indah Mohd Amin, Nando Reza Pratama

**Affiliations:** 1 Faculty of Medicine, Medical Program, Universitas Airlangga, Surabaya 60132, Indonesia; jonathan.samuel.matogu-2024@fk.unair.ac.id (J.S.M.T.); azizah.bonitha.zahrah-2024@fk.unair.ac.id (A.B.Z.S.); anindita.azkia.fauzana-2024@fk.unair.ac.id (A.A.F.); pramudya.dhafa.hernandi-2024@fk.unair.ac.id (P.D.H.); albert.even.purnama-2024@fk.unair.ac.id (A.S.P.); 2 Department of Physiology and Medical Biochemistry, Faculty of Medicine, Universitas Airlangga, Surabaya 60132, Indonesia; l.agung.waskito@fk.unair.ac.id; 3 Institute of Tropical Disease, Universitas Airlangga, Surabaya 60115, Indonesia; 4 Cardiovascular and Cardiometabolic Science Research Group, Faculty of Medicine, Universitas Airlangga, Surabaya 60132, Indonesia; hendrisusilo@staf.unair.ac.id; 5 Cardiovascular and Cardiometabolic Science Research Group, Department of Cardiology and Vascular Medicine, Universitas Airlangga Hospital, Surabaya 60115, Indonesia; 6 Cardiology and Vascular Medicine Department, Dr. Soetomo General Hospital, Surabaya 60286, Indonesia; 7 Department of Internal Medicine, Universitas Airlangga Hospital Surabaya, Surabaya 60115, Indonesia; 8 Oncology and Cancer Research Group, Faculty of Medicine, Universitas Airlangga, Surabaya 60132, Indonesia; 9 Faculty of Dentistry, Universiti Teknologi MARA, Selangor 47000, Malaysia; indahma@uitm.edu.my; 10 Nuffield Department of Medicine, University of Oxford, Oxford OX3 7BN, UK; nando.reza@sid-indonesia.org; 11 Summit Institute for Development Indonesia, Mataram 83238, Indonesia

**Keywords:** acute coronary syndrome, biomarkers, cardiovascular diseases, percutaneous coronary intervention, sST2, GDF-15, oxidative stress, prognosis

## Abstract

Acute coronary syndrome patients undergoing percutaneous coronary intervention remain at high risk for major adverse cardiovascular events (MACE: cardiovascular mortality, non-fatal myocardial infarction, and stroke). Inflammatory–oxidative stress biomarkers are potential prognostic tools; however, the influence of sampling timing—pre-procedural versus post-procedural—remains unclear. This meta-analysis evaluated six biomarkers: sST2, GDF-15, OPG, sLOX-1, H-FABP, and Galectin-3. Pooled Hazard Ratios (HRs) for time-to-event outcomes and Standardized Mean Differences (SMDs) between event and non-event groups were synthesized using random-effects models involving 40 studies (18,933 patients). Elevated pre-procedural levels of sST2 (HR = 3.32, *p* < 0.0001), GDF-15 (HR = 3.00, *p* < 0.0001), sLOX-1 (HR = 2.61, *p* = 0.0023), and OPG (HR = 1.79, *p* = 0.0206) significantly predicted MACE. Notably, pre-PCI sST2 strongly predicted heart failure hospitalization (HR = 6.30, *p* < 0.0001). Additionally, pre-PCI H-FABP demonstrated a moderate significant effect on adverse outcomes (SMD = 0.67, *p* < 0.0001). While pre-PCI Galectin-3 was not significant, its post-procedural levels showed a large significant effect (SMD = 1.15, *p* < 0.0001). In conclusion, inflammatory and oxidative stress biomarkers, particularly sST2 and GDF-15, demonstrate consistent associations with adverse outcomes in ACS patients undergoing PCI, offering more reliable baseline risk stratification than post-procedural measurements.

## 1. Introduction

Cardiovascular diseases (CVDs) remain the leading cause of mortality worldwide, accounting for approximately 19.8 million deaths in 2022, representing about 32% of all global deaths, with nearly 85% of these deaths caused by ischemic heart disease and stroke [1]. Among these conditions, acute coronary syndrome (ACS), which includes ST-segment elevation myocardial infarction (STEMI), non-ST-segment elevation myocardial infarction (NSTEMI), and unstable angina, represents a major contributor to emergency hospitalizations and cardiovascular mortality globally [2]. Currently, percutaneous coronary intervention (PCI) is considered the gold standard revascularization strategy for patients with ACS because it effectively restores coronary blood flow and improves clinical outcomes when performed promptly. However, despite advances in PCI techniques and stent technology, post-procedural complications remain a significant clinical concern. Previous studies have reported that approximately 5.1% to 17.9% of patients undergoing PCI still experience major adverse cardiovascular events (MACE) within the first year, including cardiac death, myocardial infarction, and target lesion revascularization, highlighting the need for improved risk stratification to identify high-risk patients and optimize long-term outcomes [3].

The pathophysiological progression of ACS and the subsequent response to PCI are heavily influenced by the complex interplay of systemic inflammation, oxidative stress, and myocardial remodeling. In this context, the six biomarkers investigated in this study serve as critical indicators of these pathological pathways.

Soluble suppression of tumorigenicity 2 (sST2) and Galectin-3 are primarily associated with myocardial strain and fibrosis. sST2 is a soluble variant of the interleukin-1 receptor family that normally acts as a decoy receptor to modulate the cardioprotective effects of IL-33, preventing excessive inflammatory responses in the heart [4]. Galectin-3 is a β-galactoside-binding lectin primarily secreted by activated macrophages; under normal physiological conditions, it regulates cell adhesion, proliferation, and tissue repair [5]. In ACS patients, elevated levels of these proteins reflect the heart’s maladaptive response to ischemic injury, which often leads to heart failure and long-term adverse events [4,5].

Growth differentiation factor-15 (GDF-15) and osteoprotegerin (OPG) represent systemic inflammatory stress and vascular calcification pathways, respectively. GDF-15 is a stress-induced cytokine belonging to the transforming growth factor-beta (TGF-β) superfamily, which normally functions to maintain cellular homeostasis and limit tissue damage during inflammatory stress [6]. OPG is a member of the tumor necrosis factor (TNF) receptor superfamily that acts as a decoy receptor for RANKL, a role that is crucial for maintaining normal bone metabolism and protecting the vascular media from calcium deposition [7]. In this study, they act as markers for advanced atherosclerotic burden and systemic vulnerability [6,7].

Furthermore, soluble lectin-like oxidized low-density lipoprotein receptor-1 (sLOX-1) and heart-type fatty acid-binding protein (H-FABP) are potent indicators of acute oxidative stress and rapid myocardial injury. sLOX-1 is the cleaved extracellular domain of the lectin-like oxidized low-density lipoprotein receptor-1 (LOX-1) found on endothelial cells, which normally facilitates the endocytosis of degraded LDL [8]. H-FABP is a small cytoplasmic protein that plays a vital role in the normal heart by transporting long-chain fatty acids to the mitochondria for energy production [9]. sLOX-1 plays a key role in plaque rupture through the uptake of oxidized LDL, while H-FABP is a highly sensitive marker for early myocardial necrosis due to its rapid release from damaged myocytes, making them valuable for predicting immediate post-procedural outcomes [8,9].

Although these biomarkers represent diverse pathological processes, their prognostic utility in clinical practice remains a subject of debate. One significant source of inconsistency is the timing of biomarker measurement. Most existing research evaluates biomarkers at a single timepoint, yet the prognostic value may differ significantly between pre-procedural levels, which reflect the patient’s baseline risk and plaque stability, and post-procedural levels, which may capture the extent of peri-procedural myocardial injury induced by the PCI itself [10]. Currently, there is a lack of comprehensive evidence directly comparing the predictive accuracy of these biomarkers at different stages of the PCI procedure. To date, no comprehensive synthesis has systematically compared the prognostic value of inflammatory and oxidative stress biomarkers measured before versus after PCI in ACS patients. Clarifying whether baseline or post-intervention biomarker assessment provides superior risk stratification is essential for optimizing their potential clinical application.

Therefore, this systematic review and meta-analysis aim to evaluate the prognostic utility of six key inflammatory and oxidative stress biomarkers, namely sST2, GDF-15, OPG, sLOX-1, H-FABP, and Galectin-3, in predicting adverse cardiovascular outcomes among ACS patients undergoing PCI. Specifically, we compare the predictive performance of biomarkers measured in the pre-procedural and post-procedural phases to determine the influence of sampling timing on prognostic accuracy.

## 2. Methods

### 2.1. Study Design

This systematic review was conducted and reported in accordance with the PRISMA 2020 statement (Supplementary Table S1) and the CHARMS checklist (Supplementary Table S2). The inclusion criteria were structured based on the PECO (Population, Exposure, Comparator, Outcome) framework, with additional specifications for Timing and Setting:

- Population: Adult patients (≥18 years) diagnosed with acute coronary syndrome (ACS) who underwent percutaneous coronary intervention (PCI).
- Exposure (E): Elevated concentrations of six specific inflammatory and oxidative stress biomarkers: sST2, GDF-15, OPG, sLOX-1, H-FABP, and Galectin-3. “Elevated” is defined by study-specific thresholds, such as the highest quartile or tertile or a predefined clinical cut-off point.
- Comparator: Baseline or lower concentrations of the respective biomarkers (e.g., lower quartiles/tertiles or levels below the clinical cut-off) within the same study population.
- Outcome: Prognostic endpoints including major adverse cardiovascular events (MACE), MACCE, cardiovascular mortality, all-cause mortality, heart failure hospitalization, and recurrent ACS.
- Timing: Biomarker sampling was stratified into two specific timeframes, pre-procedural (baseline) and post-procedural (acute phase following PCI), with no restriction on follow-up duration.
- Setting: Cohort, case–control, or cross-sectional studies conducted in clinical settings involving ACS patients managed with PCI.

This review aimed to comprehensively evaluate the prognostic utility of these inflammatory and oxidative stress biomarkers in predicting adverse outcomes in the ACS-PCI population. The study protocol was registered in the International Prospective Register of Systematic Reviews (PROSPERO) under the registration number CRD420251246532. A major amendment was made to the original protocol to include pre-procedural biomarkers in addition to post-procedural ones, to provide a more comprehensive prognostic assessment.

### 2.2. Search Strategy

Comprehensive electronic searches were conducted across eight major databases: PubMed, Scopus, Web of Science, ScienceDirect, ProQuest, Sage, Taylor & Francis, and Springer. Additionally, to minimize publication bias and capture the gray literature, preprint servers including BioRxiv and MedRxiv were also screened. The search was performed for studies published from inception up to 11 January 2026.

Two independent assessors (J.S.M.T. and A.B.Z.S.) performed the search and initial screening. To maximize the retrieval of relevant studies, Medical Subject Headings (MeSH) terms and free-text keywords were combined using Boolean operators (“AND”, “OR”) based on the following concepts:

- Biomarkers: “soluble lectin-like oxidized low-density lipoprotein receptor-1” OR “sLOX-1” OR “soluble LOX-1” OR “Heart-type Fatty Acid-Binding Protein” OR “H-FABP” OR “FABP3” OR “Osteoprotegerin” OR “OPG” OR “Soluble ST2” OR “sST2” OR “Growth Differentiation Factor-15” OR “GDF-15” OR “Galectin-3”.
- Disease: “Acute Coronary Syndrome” OR “ACS” OR “Myocardial Infarction” OR “STEMI” OR “NSTEMI” OR “Unstable Angina”.
- Intervention: “Percutaneous Coronary Intervention” OR “PCI.”
- Prognosis and Outcomes: “Prognosis” OR “Major Adverse Cardiac Events” OR “MACE” OR “Mortality” OR “Death” OR “Rehospitalization” OR “Recurrence.”

The specific search strings for each database are detailed in Supplementary Table S3. All retrieved records were imported into the Rayyan AI (web-based version, Rayyan Systems Inc., Cambridge, MA, USA; https://www.rayyan.ai/, accessed on 22 January 2026) for systematic reviews, where duplicates were automatically detected and resolved. The titles and abstracts were independently screened by two reviewers (J.S.M.T. and A.B.Z.S.) using the blind mode in Rayyan, followed by a full-text assessment of potentially eligible articles. Any disagreements regarding study inclusion were resolved through discussion or adjudication by a third reviewer (A.A.F.).

### 2.3. Eligibility Criteria

This systematic review included original, peer-reviewed studies involving adult human participants (≥18 years) diagnosed with acute coronary syndrome (ACS) and treated with percutaneous coronary intervention (PCI). Case identification for ACS was based on established clinical diagnostic guidelines, such as European Society of Cardiology (ESC) or American College of Cardiology (ACC)/American Heart Association (AHA) as reported in the included primary studies, ensuring consistency in patient selection. The search was limited to articles published in the English language and available in full text.

Eligible studies employed observational designs, primarily prospective or retrospective cohorts and case–control studies, that investigated the prognostic value of specific inflammatory and oxidative stress biomarkers, namely sLOX-1, H-FABP, OPG, sST2, GDF-15, and Galectin-3. To capture the temporal dynamics of the expression of biomarkers, studies providing data at either pre-procedural (baseline) or post-procedural (acute phase) timepoints were considered eligible. Inclusion was further contingent on the availability of quantitative data: specifically, studies reporting mean and standard deviation (SD) values for event vs. non-event groups or those providing categorical Hazard Ratios (HRs) with 95% confidence intervals (CIs).

Studies were excluded if they were review articles, case reports, case series, meta-analyses, or conference abstracts lacking full peer-reviewed data. Duplicate records and studies with incomplete or insufficient data for extraction were also removed. To ensure consistency between the review question and analysis, the eligibility criteria were determined following the PICOTS framework.

A standardized data extraction form was developed to ensure clarity, consistency, and comprehensiveness towards all eligible studies. Two reviewers (J.S.M.T. and A.B.Z.S.) independently performed data extraction for each study, collecting information on study characteristics (author, year, design, and location), population demographics, inflammatory marker values, sampling timing, clinical outcomes (MACE and mortality), follow-up duration, disease spectrum, measurement units, and effect estimates. Any discrepancies in extracted data were resolved through discussion between the two reviewers. If consensus could not be reached, a third reviewer (A.A.F.) was consulted for adjudication.

### 2.4. Definition of Clinical Outcomes and Sampling Timepoints

For the purpose of this meta-analysis, the primary endpoint was major adverse cardiovascular events (MACE), defined as a composite of cardiovascular mortality, non-fatal myocardial infarction (MI), and stroke, consistent with the definitions provided in the included primary studies. Secondary endpoints included Major Adverse Cardiac and Cerebrovascular Events (MACCE), all-cause mortality, cardiovascular death, heart failure (HF) hospitalization, and recurrent ACS/AMI [11].

Biomarker sampling timing was stratified into two distinct phases to evaluate temporal prognostic value:

- Pre-procedural (Pre-PCI): Defined as blood samples collected at hospital admission or immediately prior to the start of the PCI procedure (baseline).
- Post-procedural (Post-PCI): Defined as blood samples collected immediately after the procedure with no restriction on follow-up duration to capture the peri-procedural inflammatory response.

### 2.5. Quality Assessment and Certainty of Evidence

The methodological quality of the included observational studies was evaluated using the Newcastle–Ottawa Scale (NOS). This scale assesses studies across three domains: the (1) selection of study groups (maximum 4 stars); (2) comparability of the groups (maximum 2 stars); and (3) ascertainment of the outcome of interest (maximum 3 stars). Studies achieving a total score of ≥7 were considered high-quality, those with scores of 4–6 were classified as moderate-quality, and those with scores < 4 were deemed low-quality (high risk of bias) [12]. Two reviewers (J.S.M.T. and A.B.Z.S.) independently assessed quality, with any discrepancies resolved through discussion or consultation with a third reviewer (A.A.F.).

The certainty of the body of evidence for each outcome was assessed using the GRADE (Grading of Recommendations Assessment, Development and Evaluation) framework, adapted for prognostic factor research [13]. Given that well-designed prospective observational studies represent the gold standard for prognostic questions where randomization is not feasible, the initial certainty level for the included cohort studies was set to “high”.

The evidence was subsequently downgraded based on five limitations: risk of bias, inconsistency (heterogeneity I^2^ > 50%), indirectness, imprecision (wide confidence intervals), and publication bias. Conversely, the certainty of evidence could be upgraded if the magnitude of the effect was large (Hazard Ratio > 2 or SMD > 0.8) or very large (HR > 5 or SMD > 1.2) or if a clear dose–response gradient was observed.

### 2.6. Data Synthesis and Statistical Analysis

Meta-analysis was performed to synthesize pooled Standardized Mean Differences (SMDs) for continuous outcomes and pooled Hazard Ratios (HRs) for time-to-event outcomes based on the elevated inflammatory biomarker value. To ensure standardized and reproducible variable selection, quantitative meta-analysis (pooling) was only performed for biomarkers with quantitative data (SMD or HR) reported by at least two independent studies for each specific timepoint and measurement unit. Biomarkers that did not meet this threshold were instead synthesized qualitatively to provide a comprehensive prognostic overview. Categorical adjusted Hazard Ratios (aHRs) were extracted, along with their 95% confidence intervals (CIs), to rigorously account for potential covariates. For continuous variables reported as medians with ranges or interquartile ranges, means and standard deviations were estimated using the method described by Wan et al. [14], under the assumption of normal distribution. The magnitude of the SMD effect was interpreted according to Cohen’s criteria: 0.2–0.5 as small, 0.5–0.8 as moderate, and >0.8 as large [15].

Heterogeneity between studies was evaluated using the Q-test and I^2^ statistic. To ensure robust estimates, the Restricted Maximum Likelihood (REML) estimator was employed for all random-effects models, regardless of the I^2^ value, to better account for between-study variance. Forest plots were generated to visually display the effect sizes and 95% confidence intervals of individual studies and pooled analyses. Statistical analysis was carried out using the metafor package within R version 4.5.2 (R Foundation for Statistical Computing, Vienna, Austria) on a Windows x64 platform. The RStudio IDE version 2025.09.2+418 (Posit Software, PBC, Boston, MA, USA) was utilized as the development environment to ensure the reproducibility of the computational workflow.

Subgroup analyses were carried out according to study location (stratified by continent: Asian, European, and multicenter), clinical outcome (MACE, MACCE, CVD, all-cause mortality, and HF hospitalization), follow-up duration (short-term: t < 1 year; medium-term: 1 year ≤ t < 2 years; and long-term: t ≥ 2 years), and disease spectrum (STEMI, NSTEMI, and unstable angina). Publication bias was assessed quantitatively using Egger’s test for outcomes involving ≥10 studies (*p* < 0.05 considered indicative of bias). Sensitivity analysis was performed by excluding studies with a high risk of bias or low quality (NOS score < 7) to determine the consistency of the results.

## 3. Results

### 3.1. Study Selection

**Figure 1:**
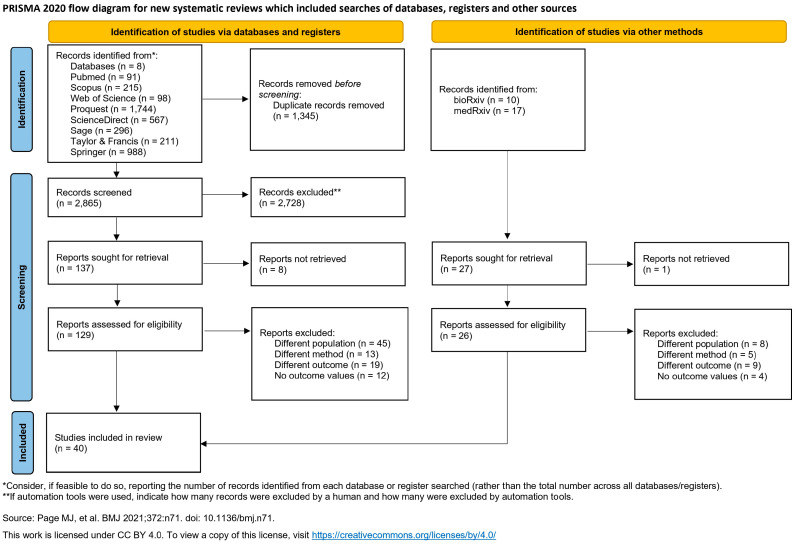
PRISMA flow diagram. Source: [16].

The comprehensive search across eight databases initially identified 4210 records. After removing duplicates and screening titles and abstracts, full-text reviews were conducted. Ultimately, 40 studies met the inclusion criteria and were included in the systematic review and meta-analysis. The primary reasons for exclusion were disparate populations, incompatible study methods, or lack of specific outcome data. A detailed list of these excluded studies, along with the specific reasons for their exclusion, is provided in Supplementary Table S4. The detailed study selection process is illustrated in the PRISMA flow diagram (Figure 1).

### 3.2. Study Characteristics

The baseline characteristics of the included studies are summarized in Table 1. A total of 18,933 patients with acute coronary syndrome (ACS) undergoing PCI were analyzed across the 40 included studies. Geographically, most studies were conducted in Asia (*n* = 19) and Europe (*n* = 17), with fewer studies from America (*n* = 1) and multicenter collaborations (*n* = 3).

Regarding the specific biomarkers, sST2 was the most frequently studied marker (16 studies), followed by GDF-15 (9 studies), Galectin-3 (7 studies), sLOX-1 (4 studies), and H-FABP (3 studies). Notably, four studies [17,18,19,20] evaluated multiple biomarkers simultaneously. In terms of sampling timing, the majority of studies (*n* = 29) focused on pre-PCI measurements, while six studies assessed post-PCI levels, and five studies provided data for both timepoints.

For statistical synthesis, 25 studies provided sufficient data for Standardized Mean Difference (SMD) calculations, while 21 studies reported Hazard Ratios (HRs). The follow-up duration varied widely: short-term in 9 studies, medium-term in 18 studies, and long-term in 13 studies. The reported clinical endpoints were diverse, comprising MACE (*n* = 13), all-cause mortality (*n* = 6), cardiovascular disease (CVD) events (*n* = 6), death or heart failure rehospitalization (*n* = 6), MACCE (*n* = 5), and ACS/AMI recurrence (*n* = 4).

**Table 1:** Characteristics of included studies.

Author (Year)	Marker	Study Location	Population Age	Sample Size		Disease Spectrum	PCI Rate (%)	Clinical Outcome	Sampling Timepoint	Follow-Up	Biomarker Concentration (Mean ± SD)		Hazard Ratio (95% CI)	Measurement Unit
				Events	Non-Events						Events	Non-Events		
Higuma (2015) [21]	sLOX-1	Japan	67 ± 12	20	133	STEMI	94.1%	MACE	Pre-PCI	3 years	N/A	N/A	3.46 (1.16–10.27)	ng/mL
Zhao (2019) [22]	sLOX-1	China	MACCE: 67 (59–74) Non-MACCE: 69 (59–76)	115	869	Mixed (78.05% ACS)	100%	MACCE	Pre-PCI	2 years	1.48 ± 0.93	1.15 ± 0.81	N/A	ng/mL
Kumar (2021) [23]	sLOX-1	India	47.94 ± 14.30	9	123	Mixed (48.5% ACS)	100%	Stroke, recurrent myocardial infarction (MI)	Pre-PCI and post-PCI	1 year	Pre-PCI: 894.44 ± 725.25 Post-PCI: 2086.22 ± 1079.43	Pre-PCI: 790.18 ± 670.93 Post-PCI: 1332.81 ± 967.50	N/A	ng/mL
Kraler (2022) [24]	sLOX-1	Multicenter	63.7 ± 10.27	107	2.532	ACS	100%	CVD	Pre-PCI	1 year	N/A	N/A	2.29 (1.19–5.34)	ng/mL
Ishii (2005) [25]	H-FABP	Japan	64.9 ± 10.4	25	303	ACS	55.5%	CVD, non-fatal AMI	Pre-PCI	6 months	119 ± 187	46.6 ± 86.5	8.96 (2.64–30.4)	µg/L
Erdal (2023) [26]	H-FABP	Turkey	54.6 ± 12.4	26	123	STEMI	100%	CVD	Pre-PCI and post-PCI 12 h after the start of symptoms	6 years	Pre-PCI: 28.20 ± 42.00 Post-PCI: 43.00 ± 53.33	Pre-PCI: 16.33 ± 25.19 Post-PCI: 26.33 ± 43.70	N/A	ng/mL
Kopp (2025) [17]	H-FABP	Russia	63.0 (53.5–70.0)	18	147	STEMI	97.8%	CVD	Pre-PCI	3 years	5.32 ± 10.81	1.43 ± 2.67	N/A	ng/mL
	sST2										15.53 ± 9.47	8.63 ± 12.52		
Canga (2012) [27]	OPG	Turkey	MACE: 66 ± 10 tahun Non-MACE: 60 ± 13 tahun	18	78	STEMI	100%	MACE	Pre-PCI	In hospital	215 ± 257	85 ± 66	N/A	pg/mL
Hyseni (2013) [28]	OPG	Netherlands and Germany	Survivors = 67.5 (59.5–73.4) Non-survivors = 76.8 (68.9–83.4)	46	293	ACS	100%	All-cause mortality	Pre-PCI	4 years	N/A	N/A	1.56 (0.75–3.24)	μg/mL
Bjerre (2014) [29]	OPG	Denmark	Low OPG = 60.3 ± 10.5 High OPG = 66.9 ± 10.8	47	172	STEMI	100%	MACCE	Pre-PCI	45 (38–48) months	N/A	N/A	2.0 (1.03–3.89)	ng/L
Fuernau (2014) [18]	OPG	Germany	71 (58–79)	78	112	AMI with cardiogenic shock	95.8%	All-cause mortality	Immediate post-PCI	30 days	1274.33 ± 1547.41	690.33 ± 627.41	N/A	ng/L
	GDF-15						100%				10,590.67 ± 6217.78	6327.33 ± 5382.96		
Lindberg (2014) [30]	OPG	Denmark	60 ± 13	3	39	STEMI with total occlusion of the left coronary artery branch	100%	All-cause mortality, HF hospitalization	Pre-PCI	1 year	5401.67 ± 5540.74	2630.33 ± 335.56	N/A	ng/L
Wang (2017) [31]	sST2	China	61.41 ± 8.90	59	121	AMI	100%	MACE	Pre-PCI	1 year	823.47 ± 149.25	601.83 ± 125.84	N/A	ng/mL
Yu (2017) [32]	sST2	South Korea	59.1 ± 13.1	38	285	STEMI	100%	MACCE	Pre-PCI	1 year	84.0 ± 29.7	74.7 ± 27.6	2.098 (1.008–4.367)	ng/mL
Huang (2018) [33]	sST2	China	68.5 (30–72)	21	157	STEMI	71.9%	MACE	Pre-PCI	1 year	N/A	N/A	2.653 (1.201–8.929)	ng/mL
Liu (2019) [34]	sST2	China	60.2 ± 10.8	19	276	STEMI	100%	MACE	Pre-PCI	1 year	N/A	N/A	2.23 (1.20–6.78)	ng/mL
Tyminska (2019) [19]	sST2	Poland	61.0 (50.5–67.0)	9	95	STEMI	100%	CVD, HF hospitalization	Post-PCI 72–96 h after admission	1 year	N/A	N/A	11.79 (1.52–91.26)	ng/mL
	Galectin-3												14.51 (1.46–143.95)	ng/mL
Somuncu (2020) [35]	sST2	Turkey	Low sST2: 60.0 ± 12.1 High sST2: 60.5 ± 11.6	68	312	AMI	75%	MACE	Pre-PCI	1 year	N/A	N/A	2.263 (1.124–4.557)	ng/mL
Zagidullin (2020) [36]	sST2	Russia	60.9 ± 12.1	33	114	STEMI	88.4%	CVD	Pre-PCI	2 years	93.7 ± 97.1	51.3 ± 47.3	3.88 (1.27–11.84)	ng/mL
Hongisto (2021) [20]	sST2	Multicenter	81 ± 4	26	30	Mixed (80.82% ACS with cardiogenic shock)	69.64%	All-cause mortality	Post-PCI 12 h after admission	In hospital	1008.00 ± 763.70	572.00 ± 453.33	N/A	ng/mL
	GDF-15										27,276.00 ± 24,171.11	8040.67 ± 6968.89		ng/L
Zhang (2021) [37]	sST2	China	Low sST2: 61.13 ± 9.67 High sST2: 63.71 ± 10.02	25	180	NSTEMI, UA	100%	MACCE	Pre-PCI	1 year	N/A	N/A	10.22 (4.05–25.7)	ng/mL
Liu (2022) [38]	sST2	China	62 (55–70)	73	277	STEMI	100%	MACE	Pre-PCI and post-PCI 24 h after admission	1 year	Pre-PCI: 8.30 ± 9.63 Post-PCI: 17.07 ± 19.19	Pre-PCI: 10.33 ± 13.33 Post-PCI: 13.97 ± 16.96	N/A	ng/mL
Mechtouff (2022) [39]	sST2	France	59 ± 12	25	226	STEMI	100%	MACE	Pre-PCI and post-PCI 24 h after admission	1 year	Pre-PCI: 16.90 ± 7.78 Post-PCI: 40.00 ± 32.96	Pre-PCI: 13.00 ± 6.22 Post-PCI: 25.70 ± 17.19	Post PCI: 2.4 (1.03–5.5)	ng/mL
Che (2025) [40]	sST2	China	60.77 ± 12.61	37	186	STEMI	100%	MACCE	Pre-PCI	630 ± 433 days	74.41 ± 41.10	48.67 ± 23.80	4.038 (2.077–7.852)	ng/mL
Lam (2025) [41]	sST2	Vietnam	70.35 ± 11.50	16	19	STEMI	100%	Mortality, HF	Pre-PCI	30 days	79.47 ± 62.79	26.18 ± 12.97	N/A	ng/mL
Xu (2025) [42]	sST2	China	MACE: 65.26 ± 14.54 Non-MACE: 59.16 ± 12.81	39	145	STEMI	100%	MACE	Pre-PCI and post-PCI 24 h after admission	6 months	Pre-PCI: 39.10 ± 29.24 Post-PCI: 31.63 ± 27.99	Pre-PCI: 18.18 ± 11.80 Post-PCI: 47.40 ± 39.30	N/A	ng/mL
Yin (2025) [43]	sST2	China	MACE: 60.54 ± 11.17 Non-MACE: 57.73 ± 7.62	59	376	NSTEMI	100%	MACE	Pre-PCI	1 year	67.46 ± 24.46	51.23 ± 27.57	3.35 (1.894–5.914)	ng/mL
Eitel (2011) [44]	GDF-15	Germany	67 (56–75)	20	218	STEMI	100%	All-cause mortality	Pre-PCI	6 months	3071.00 ± 2170.37	1346.67 ± 632.59	N/A	ng/L
Velders (2015) [45]	GDF-15	Multicenter	59 (IQR: 15)	345	5040	STEMI	100%	CVD	Pre-PCI	282 (IQR:180) days	N/A	N/A	2.27(1.32–4.09)	ng/L
Sun (2018) [46]	GDF-15	China	CIN: 71.6 ± 13.0 Non-CIN: 63.8 ± 11.7	N/A	N/A	AMI	100%	MACE	Pre-PCI	30 days	N/A	N/A	3.562 (1.658–7.652)	ng/L
Bodde (2019) [47]	GDF-15	Netherlands	59.0 ± 11.5	37	253	STEMI	100%	All-cause mortality	Pre-PCI	9.4 (8.8–10.0) years	N/A	N/A	2.453 (1.064–5.658)	pmol/L
Peiro (2019) [48]	GDF-15	Spain	64.8 (55.6–74.3)	101	257	ACS	69.3%	MACE	Pre-PCI or peri-procedural	4.9 (4.2–5.8) years	N/A	N/A	2.48 (1.41–4.34)	ng/L
Gurgoze (2023) [49]	GDF-15	Netherlands	62.5 (54.3–70.2)	45	799	ACS	86%	CVD, recurrent ACS	Pre-PCI	1 year	2285.33 ± 1783.70	1368.00 ± 697.78	N/A	pg/mL
Peiro (2025) [50]	GDF-15	Spain	NHRHF: 59.7 (51.4–69.3) HRHF: 72.7 (64.0–79.2)	49	226	AMI	74.18%	All-cause mortality, HF hospitalization	Pre-PCI or peri-procedural	Median 4.9 years	N/A	N/A	6.3 (2.9–13.3)	ng/L
Lisowska (2016) [51]	Galectin-3	Poland	STEMI: 63.3 ± 9.9 NSTEMI: 63.5 ± 11.8	21	212	STEMI, NSTEMI	100%	All-cause mortality	Pre-PCI	Mean 2.8 years	19.57 ± 11.04	8.13 ± 3.26	N/A	ng/mL
Di Tano (2017) [52]	Galectin-3	Italy	65 (56–76)	20	83	STEMI	100%	All-cause mortality, HF hospitalization	Post-PCI 24 h after admission	22 (3–30) months	26.60 ± 18.59	12.77 ± 3.19	N/A	ng/mL
Mosleh (2018) [53]	Galectin-3	United States	60.94 ± 13.53	12	69	STEMI	100%	MACE	Post-PCI 12–48 h after admission	1 year	19.07 ± 11.10	11.75 ± 5.64	N/A	ng/mL
Gagno (2019) [54]	Galectin-3	Italy	67.63 ± 11.29	35	434	STEMI, NSTEMI	70.6%	CVD	Pre-PCI	1 year	12.11 ± 4.33	9.88 ± 3.33	N/A	ng/mL
Swiecki (2020) [55]	Galectin-3	Poland	STEMI: 62.4 ± 9.7 NSTEMI: 64.2 ± 10.1	67	43	STEMI, NSTEMI	100%	Cardiac disorder rehospitalization	Post-PCI 24 h after admission	41.3 ± 9.6 months	13.7 ± 7.3	8.5 ± 4.5	N/A	ng/mL
Choi (2025) [56]	Galectin-3	South Korea	Low Galectin-3: 60.7 ± 10.0 High Galectin-3: 65.0 ± 10.8	92	847	Mixed (51.18% ACS, 43.77% SA, 5.22% SI)	100%	All-cause mortality, AMI, stroke	Pre-PCI	997 (766–1.264) days	N/A	N/A	1.670 (1.014–2.751)	ng/mL

Abbreviations: (1) STEMI: ST-Elevation Myocardial Infarction; (2) NSTEMI: Non-ST-Elevation Myocardial Infarction; (3) UA: Unstable Angina; (4) ACS: Acute Coronary Syndrome (STEMI, NSTEMI, UA); (5) AMI: Acute Myocardial Infarction (STEMI and NSTEMI); (6) MACE: Major Adverse Cardiovascular Events; (7) MACCE: Major Adverse Cardiovascular and Cerebrovascular Events; (8) CVD: Cardiovascular Death; (9) CIN: Contrast-Induced Nephropathy; (10) HF: Heart Failure; (11) HRHF: High Risk of Development of HF; (12) NHRHF: No High Risk of Development of HF; (13) MI: Myocardial Infarction; (14) SA: Stable Angina; (15) SI = Silent Ischemia.

### 3.3. Methodological Quality of Included Studies

The methodological quality of the included cohort studies was rigorously evaluated using the Newcastle–Ottawa Scale (NOS). The complete assessment scores for each study are detailed in Supplementary Table S5. Overall, the quality of the included literature was high. Out of 40 studies, 38 studies (95%) were classified as high-quality (score ≥ 7), indicating a low risk of bias. Only two studies [23,25] were classified as moderate-quality (score of 6).

In the Selection domain, the majority of studies demonstrated an excellent representativeness of the exposed cohort and accurate ascertainment of exposure. Regarding the Comparability domain, most studies successfully adjusted for key confounding factors such as age and comorbidities (indicated by two stars), although a few studies lacked comprehensive multivariate adjustment. In the Outcome domain, follow-up duration and the adequacy of follow-up cohorts were generally sufficient to capture major adverse events, though some studies lost points due to a lack of description regarding lost-to-follow-up rates.

### 3.4. Quantitative Data Synthesis

#### 3.4.1. Biomarker Levels and Clinical Outcomes (SMD Analysis)

**Figure 2:**
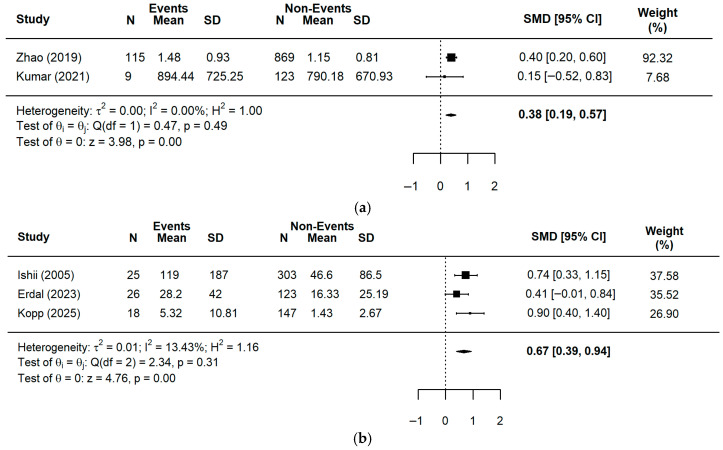

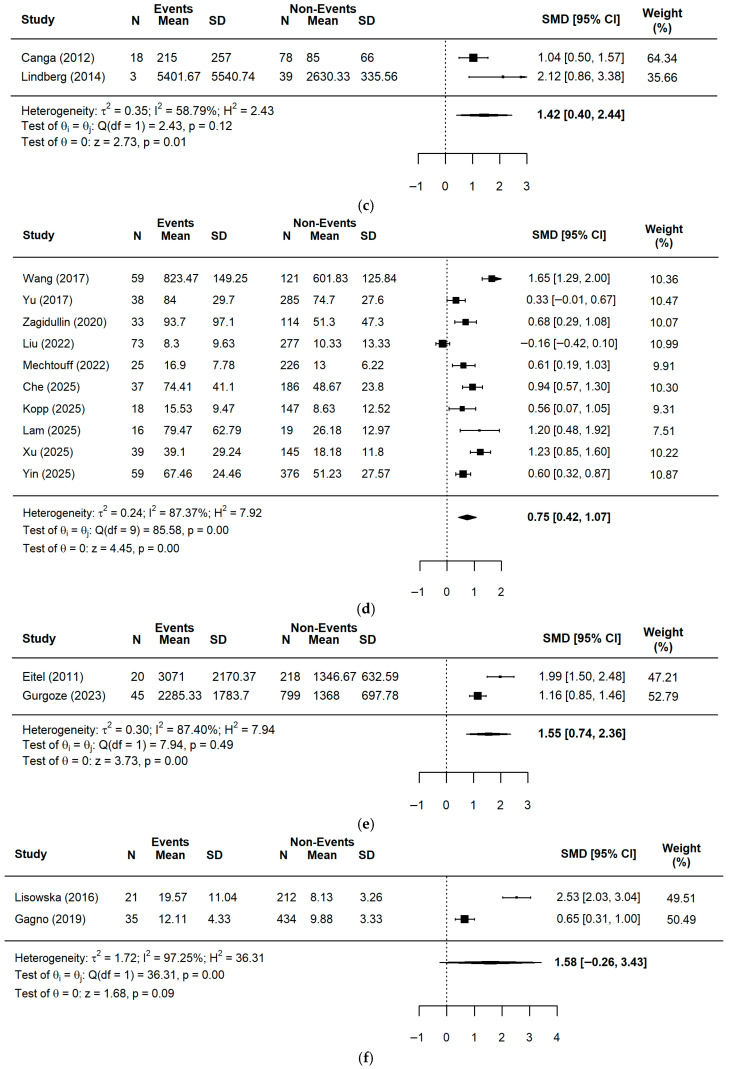
A forest plot for the pooled SMD in events and non-events among ACS patients pre-PCI: (**a**) sLOX-1 [22,23]; (**b**) H-FABP [17,25,26]; (**c**) OPG [27,30]; (**d**) sST2 [17,31,32,36,38,39,40,41,42,43]; (**e**) GDF-15 [44,49]; (**f**) Galectin-3 [51,54].

The meta-analysis using the Standardized Mean Difference (SMD) demonstrated varying prognostic effects across biomarkers and the timing of sample collection. Regarding pre-procedural measurements (Figure 2), elevated levels of sLOX-1 (2 studies, *n* = 1116) demonstrated a small but significant effect on adverse clinical outcomes (SMD = 0.38, 95% CI = 0.19–0.57, *p* < 0.0001), while H-FABP (3 studies, *n* = 642) and sST2 (10 studies, *n* = 2293) showed moderate and significant effects with SMD values of 0.67 and 0.75, respectively (H-FABP: SMD = 0.67, 95% CI = 0.39–0.94, *p* < 0.0001; sST2: SMD = 0.75, 95% CI = 0.42–1.07, *p* < 0.0001). In contrast, pre-PCI elevations in OPG (two studies, *n* = 138) and GDF-15 (two studies, *n* = 1082) exhibited large and significant effects, reaching SMD values of 1.42 and 1.55, respectively (OPG: SMD = 1.42, 95% CI = 0.40–2.44, *p* = 0.0063; GDF-15: SMD = 1.55, 95% CI = 0.74–2.36, *p* = 0.0002). Notably, pre-procedural Galectin-3 (two studies, *n* = 702) did not show a statistically significant association with the incidence of major adverse events (SMD = 1.58, 95% CI = −0.26–3.43, *p* = 0.0922).

**Figure 3:**
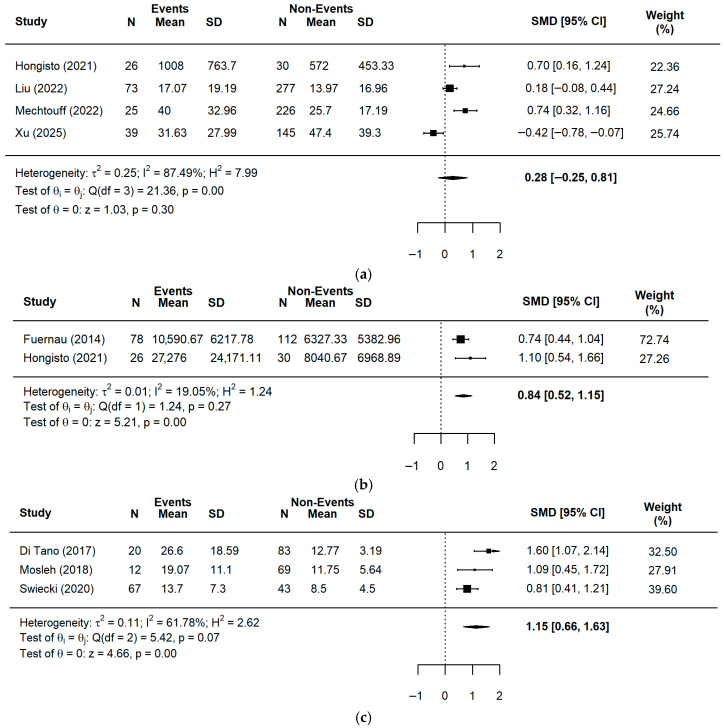
A forest plot for the pooled SMD in events and non-events among ACS patients post-PCI: (**a**) sST2 [20,38,39,42]; (**b**) GDF-15 [18,20]; (**c**) Galectin-3 [52,53,55].

For post-procedural assessments (Figure 3), both GDF-15 (two studies, *n* = 246) and Galectin-3 (three studies, *n* = 294) consistently showed large and significant effects (GDF-15: SMD = 0.84, 95% CI = 0.52–1.15, *p* < 0.0001; Galectin-3: SMD = 1.15, 95% CI = 0.66–1.63, *p* < 0.0001), whereas post-PCI sST2 (four studies, *n* = 841) levels did not show a significant association in terms of clinical outcomes (SMD = 0.28, 95% CI = −0.28–0.81, *p* = 0.3049). Meta-analyses for post-PCI sLOX-1, H-FABP, and Galectin-3 using the SMD unit were not feasible due to an insufficient number of eligible studies.

#### 3.4.2. Prognostic Value for Adverse Events (Hazard Ratio Analysis)

**Figure 4:**
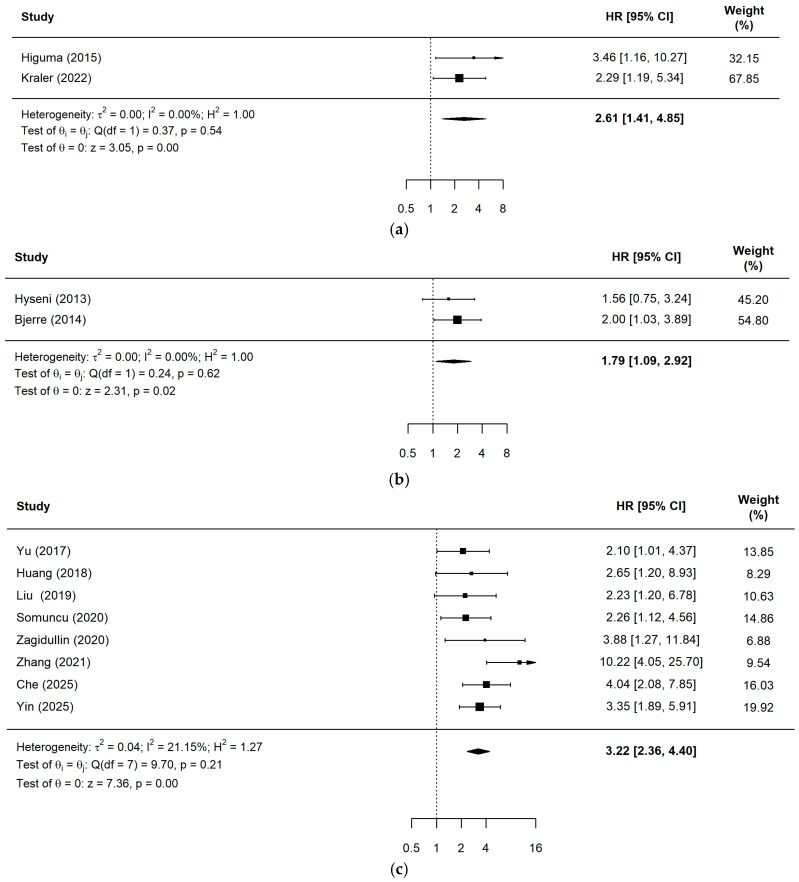

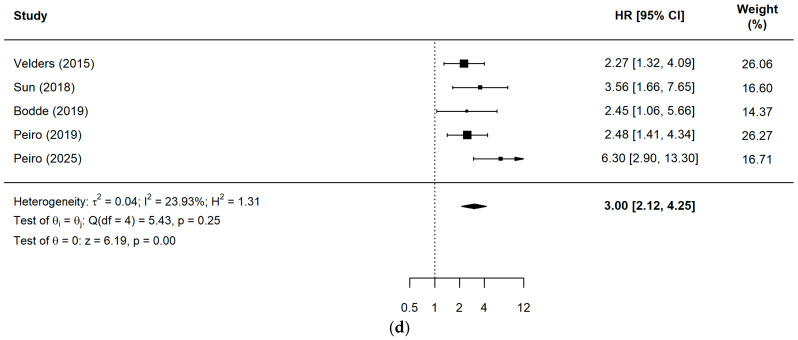
A forest plot for the pooled HR in events and non-events among ACS patients pre-PCI: (**a**) sLOX-1 [21,24]; (**b**) OPG [28,29]; (**c**) sST2 [32,33,34,35,36,37,40,43]; (**d**) GDF-15 [45,46,47,48,50].

**Figure 5:**
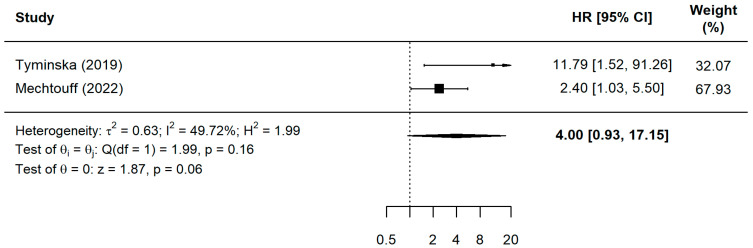
A forest plot for the sST2 pooled HR in events and non-events among ACS patients post-PCI [19,39].

The prognostic value of these biomarkers was further substantiated by the pooled Hazard Ratio (HR) analysis, which indicated a significantly increased risk of major adverse events associated with elevated inflammatory and oxidative stress markers (Figure 3). As illustrated in Figure 4, patients with high pre-PCI sLOX-1 (two studies, *n* = 2792) levels had a 2.61-fold increased risk of adverse events, while elevated pre-PCI OPG (two studies, *n* = 558) levels increased the risk by 1.79 times (sLOX-1: HR = 2.61, 95% CI = 1.41–4.85, *p* = 0.0023; OPG: HR = 1.79, 95% CI = 1.09–2.92, *p* = 0.0206). Pre-procedural sST2 (eight studies, *n* = 2186) and GDF-15 (five studies, *n* = 6619) emerged as particularly robust predictors, with risk increases of 3.32-fold and 3.00-fold, respectively (sST2: HR = 3.32, 95% CI = 2.36–4.40, *p* < 0.0001; GDF-15: HR = 3.00, 95% CI = 2.12–4.25, *p* < 0.0001). Although the analysis for post-PCI sST2 (two studies, *n* = 355) yielded a high point estimate (Figure 5), this result did not reach statistical significance (HR = 4.00, 95% CI = 0.36–4.40, *p* = 0.0621) as the confidence interval crossed the null value.

### 3.5. Qualitative Synthesis of Single Studies

Where meta-analysis was not feasible due to limited data, a qualitative assessment of single studies consistently supported the prognostic value of these markers across additional timepoints. For post-PCI measurements, Kumar et al. [23] reported substantially higher sLOX-1 levels in patients with adverse events (2086.22 ± 1079.43 ng/mL) compared to event-free patients (1332.81 ± 967.50 ng/mL). Similarly, Fuernau et al. [18] observed a nearly two-fold increase in post-PCI OPG concentrations among patients with adverse outcomes (1274.33 ± 1547.41 vs. 690.33 ± 627.41 ng/L). Regarding H-FABP, Erdal et al. [26] found elevated post-PCI levels in the event group (43.00 ± 53.33 vs. 26.33 ± 43.70 ng/mL), while Ishii et al. [25] demonstrated a profound predictive value for pre-PCI levels with a Hazard Ratio of 8.96 (95% CI: 2.64–30.4). Finally, Hazard Ratio analyses for Galectin-3 from single studies reinforced its risk prediction capability both pre-PCI (Choi et al. [56]: HR = 1.67, 95% CI: 1.01–2.75) and post-PCI (Tyminska et al. [19]: HR = 14.51, 95% CI: 1.46–143.95), suggesting that its prognostic utility may extend beyond the limits of current pooled estimates.

### 3.6. Subgroup Analysis

Among the biomarkers evaluated, sST2, GDF-15, H-FABP, and Galectin-3 provided sufficient data for detailed subgroup analyses (Table 2). For sST2, the pre-PCI levels were stratified across several categories. Based on follow-up duration, a large and statistically significant effect was observed in the short term (SMD = 1.21; *p* = 0.003) and a moderate significant effect in the medium term (SMD = 0.65; *p* = 0.003), whereas no significant effects was observed in the long term. Based on clinical outcome, a moderate and significant effect was observed for MACE (SMD = 0.77; *p* = 0.004), while other specific outcomes such as MACCE, CVD, and mortality/HF remained non-significant. Geographical subgrouping indicated a large significant effect in Asian populations (SMD = 0.80; *p* = 0.0001) but not in European populations (*p* = 0.06). Regarding disease spectrum, pre-PCI sST2 showed significant effects in patients with STEMI (SMD = 0.64; *p* = 0.0001) and AMI (SMD = 1.65; *p* = 0.0004). For post-PCI sST2 levels, the subgroup analyses by follow-up duration, clinical outcome, and geography consistently yielded non-significant associations across all categories, including short-term and medium-term follow-ups.

Regarding Hazard Ratio (HR) measurements, subgroup analyses were conducted for pre-PCI sST2 and GDF-15. For pre-PCI sST2, elevated levels significantly increased the risk of MACE (HR = 2.66; *p* < 0.0001) and MACCE (HR = 4.07; *p* < 0.0001), while the association with CVD was also significant but with a wide confidence interval (HR = 3.88; *p* = 0.03). Analysis by disease type further confirmed high risk across STEMI (HR = 2.88), NSTEMI (HR = 3.35), and AMI (HR = 2.26) populations. For pre-PCI GDF-15, subgrouping confirmed its robust prognostic value across short-term (HR = 2.74; *p* = 0.001) and long-term (HR = 3.28; *p* < 0.0001) timeframes. Significant risk increases were also observed across clinical outcomes including MACE (HR = 2.82), CVD (HR = 2.27), and all-cause mortality (HR = 2.45), with the highest risk seen in the combined mortality and HF hospitalization group (HR = 6.30; *p* < 0.0001).

Subgroup analyses were also performed for H-FABP (pre-PCI) and Galectin-3 (post-PCI) using the SMD unit. For pre-PCI H-FABP, significant effects were observed in both European (SMD = 0.90) and Asian (SMD = 0.58) populations, as well as in patients with STEMI (SMD = 0.64; *p* = 0.01). For post-PCI Galectin-3, large significant effects were maintained in European cohorts (SMD = 1.19) and in patients with STEMI (SMD = 1.37; *p* < 0.0001) and AMI (SMD = 0.81; *p* = 0.005).

### 3.7. Heterogeneity Exploration

Substantial heterogeneity (I^2^ > 50%) was observed across several analyses, suggesting further investigation into potential moderators. For pre-PCI sST2, subgroup analysis identified geographical location (QM, *p* = 0.0001) and clinical outcome (QM, *p* = 0.0035) as significant moderators contributing to the variance. Similarly, for post-PCI Galectin-3, heterogeneity was significantly explained by disease spectrum (*p* < 0.0001) and study location (*p* = 0.0021), indicating that the prognostic value of this marker is highly dependent on the specific patient population and setting.

Conversely, for post-PCI sST2 (k = 4), none of the analyzed moderators, including follow-up duration (*p* = 0.56), clinical outcome (*p* = 0.46), and geography (*p* = 0.17), yielded significant interaction *p*-values, indicating that the source of heterogeneity in the post-procedural phase was not driven by these study characteristics.

For several biomarkers with a limited number of studies, a subgroup analysis was not feasible; however, qualitative inspection showed specific drivers of variance. For pre-PCI OPG (I^2^ = 58.79%), heterogeneity was likely driven by marked differences in disease spectrum (total occlusion STEMI vs. general STEMI) and clinical endpoints (MACE vs. mortality/HF). For pre-PCI GDF-15 (I^2^ = 87.40%), the variance might be attributable to variations in disease spectrum (STEMI-only vs. general ACS) and follow-up duration (6 months vs. 1 year). Finally, for pre-PCI Galectin-3 (I^2^ = 97.25%), the considerable heterogeneity was attributed to differences in clinical outcomes (CVD vs. all-cause mortality) and follow-up length (1 year vs. mean 2.8 years) between the included studies.

### 3.8. Certainty of Evidence (GRADE)

The certainty of evidence was assessed using the GRADE framework, with observational studies initially set to high certainty (Table 3). Overall, the certainty of evidence was predominantly high, particularly for pre-PCI sLOX-1 (SMD/HR), H-FABP (SMD), OPG (HR), and post-PCI GDF-15 (SMD), where no serious limitations were identified.

Despite substantial heterogeneity (I^2^ > 50%), several outcomes maintained high certainty after being upgraded for the magnitude of effect. Specifically, pre-PCI GDF-15 and OPG (SMD) were upgraded for very large effects (SMD > 1.2), while pre-PCI sST2 (HR), GDF-15 (HR), and post-PCI Galectin-3 (SMD) were upgraded for large effects (HR > 2 or SMD > 0.8).

In contrast, the certainty of pre-PCI sST2 (SMD) was graded as Moderate due to unresolved inconsistency. Finally, the evidence for pre-PCI Galectin-3 and post-PCI sST2 (both SMD and HR) was downgraded to low-certainty, primarily due to serious imprecision where confidence intervals crossed the null value or were extremely wide.

### 3.9. Sensitivity Analysis and Publication Bias

A sensitivity analysis was conducted to assess the robustness of our findings by excluding studies classified as having moderate quality (NOS score = 6), specifically Kumar et al. [23] and Ishii et al. [25]. For pre-PCI H-FABP, the exclusion of Ishii et al. [25] resulted in a revised pooled SMD of 0.64 (95% CI: 0.16–1.11, *p* = 0.01). This slight shift from the initial estimate (SMD = 0.67, 95% CI: 0.39–0.94, *p* < 0.0001) confirms that the significant prognostic value of H-FABP remained consistent and robust even when the analysis was restricted exclusively to high-quality studies.

For pre-PCI sLOX-1, a formal sensitivity analysis via exclusion could not be performed, as removing Kumar et al. [23] would leave only a single study (Zhao et al. [22]) in the pooled analysis, precluding a recalculated meta-analysis. However, the qualitative finding from this remaining high-quality study continued to demonstrate a significant association between elevated sLOX-1 and adverse cardiovascular outcomes.

Furthermore, potential publication bias (reporting biases) was quantitatively assessed for pre-PCI sST2, as it was the only subgroup containing ≥10 studies. Egger’s test revealed no evidence of significant small-study effects or publication bias (z = 1.1633, *p* = 0.2447).

## 4. Discussion

This systematic review and meta-analysis evaluated the prognostic utility of six inflammatory and oxidative stress biomarkers—sST2, GDF-15, OPG, sLOX-1, H-FABP, and Galectin-3—in ACS patients undergoing PCI. The primary findings demonstrated that elevated pre-procedural levels of these biomarkers, particularly sST2 and GDF-15, were significantly associated with an increased risk of MACE. Notably, this study highlights a clear disparity in prognostic value based on the timing of measurement, where pre-procedural sampling generally provided a more consistent and stable prediction of long-term outcomes compared to post-procedural assessments. This consistency may be attributed to the fact that pre-procedural biomarker levels reflect the underlying chronic myocardial injury, systemic inflammation, and baseline cardiovascular risk burden, whereas post-procedural elevations may represent both baseline inflammation and the response of myocardial injury and systemic inflammation to the procedure [57].

The strong prognostic performance observed for sST2 and GDF-15 is biologically plausible with their established roles in myocardial stress and systemic inflammation. sST2 is a member of the interleukin-1 receptor family that reflects myocardial strain and fibrosis [5]. Our comprehensive subgroup analysis utilizing HRs revealed critical insights into the specific utility of sST2. Although sST2 demonstrated prognostic significance across the ACS spectrum, the magnitude of risk varied by clinical subtype. Strikingly, sST2 demonstrated particularly strong prognostic value in patients with NSTEMI and unstable angina. This finding suggests that in non-ST-elevation presentations, where myocardial necrosis can be caused by a wide spectrum of occlusion sizes with less extensive effects, inflammation is more dominant than necrosis. Hence, increasing levels of sST2 may serve as a sensitive marker of myocardial strain, inflammatory activation, and early ventricular remodeling beyond traditional markers of necrosis [58]. However, while the NSTEMI/UA subgroup showed a dramatically elevated risk (HR > 10), this estimate should be interpreted with caution due to the wide confidence intervals and the fact that it was derived from a relatively limited sample size in this specific subgroup. Nevertheless, this trend suggests that sST2 may be particularly valuable in capturing subclinical hemodynamic stress or ongoing ischemia in non-ST-elevation cases where conventional markers of necrosis might be less definitive.

Furthermore, when stratified by clinical outcome, sST2 exhibited a potential specificity for heart failure. The association between elevated sST2 levels and heart failure hospitalization was substantially stronger than that observed for composite MACE or all-cause mortality. This reinforces the biological plausibility that sST2 is a direct marker of maladaptive cardiac remodeling and fibrosis, which are the primary drivers of post-ACS heart failure. This finding is consistent with the molecular mechanism where sST2 acts as a “decoy” receptor, sequestering IL-33 and thereby abrogating its cardioprotective and anti-fibrotic effects [59]. Geographically, our analysis confirmed that sST2 remains a robust prognostic marker across both Asian and European populations, suggesting universal applicability across different ethnic and regional contexts. Interestingly, although post-PCI sST2 showed a high point estimate for adverse outcomes, this association did not reach statistical significance. Heterogeneity analysis further illuminated this finding: while variance in pre-PCI sST2 was significantly explained by geography and outcome, the heterogeneity in post-PCI sST2 could not be attributed to any measured study characteristics. This suggests that the post-procedural lack of significance may reflect acute inflammatory responses triggered by PCI itself, including endothelial injury and cytokine activation, which can transiently influence circulating biomarker levels and obscure their prognostic signal immediately after the procedure [60].

Similarly, GDF-15 emerged as a potent predictor of MACE in both pre- and post-procedural settings. As a stress-responsive cytokine, GDF-15 is upregulated in response to inflammation, oxidative stress, and tissue injury [7]. Subgroup analysis based on follow-up duration confirmed the durability of this marker, with significant associations persisting from the medium term into long-term follow-up. This consistency suggests that GDF-15 functions as an “integrative” marker of multi-pathway biological stress, making it less affected by the acute procedural fluctuations compared to other markers, supporting its utility for continuous risk assessment in ACS patients [7].

Regarding markers of acute injury and oxidative stress, sLOX-1 and H-FABP demonstrated significant, albeit varying, effects. The moderate but significant effect of pre-PCI H-FABP likely reflects its established role as an early biomarker of myocardial ischemia and necrosis [61,62]. Due to its low molecular weight and cytoplasmic localization, H-FABP is rapidly released into the circulation following myocardial injury and may become elevated within the first few hours after symptom onset, often earlier than conventional cardiac troponins [61,63]. Beyond its diagnostic utility, elevated H-FABP levels have consistently been associated with adverse short- and long-term outcomes in patients with acute coronary syndromes, including increased mortality and recurrent myocardial infarction [64]. In this context, higher pre-PCI H-FABP concentrations may reflect a greater burden of ischemic myocardial injury at presentation, thereby identifying patients at increased risk of subsequent adverse events. Accordingly, H-FABP may capture an early phase of myocardial injury that could be underrecognized by baseline troponin measurements, particularly in patients presenting shortly after symptom onset [61,62].

The prognostic relevance of sLOX-1 likely reflects its biological role as the principal receptor for oxidized low-density lipoprotein (ox-LDL) within the vascular wall. The activation of membrane-bound LOX-1 promotes endothelial dysfunction, oxidative stress, and inflammatory signaling, processes that contribute to atherosclerotic plaque vulnerability. Upon activation, LOX-1 undergoes proteolytic cleavage, releasing its soluble form (sLOX-1) into the circulation, where circulating levels are thought to reflect enhanced receptor expression in inflamed vascular tissue [65]. In contrast to cardiac troponins, which indicate established myocardial necrosis, sLOX-1 levels are markedly elevated during the acute phase of acute coronary syndrome (ACS) and may rise early in the course of ischemia [64]. Elevated sLOX-1 has been associated with adverse cardiovascular outcomes, including major adverse cardiovascular events and heart failure, suggesting that it captures a pathophysiological dimension related to plaque instability and vascular inflammation beyond myocardial necrosis alone [65]. Taken together, these findings support the concept that heightened vascular oxidative and inflammatory activation prior to or during the acute event may influence subsequent clinical outcomes and that sLOX-1 may provide prognostic information complementary to conventional necrosis markers.

In contrast, the findings for Galectin-3 present a more complex picture. While pre-PCI Galectin-3 levels were not significantly associated with adverse outcomes, post-PCI levels showed a large significant effect. This discrepancy strongly suggests that Galectin-3 kinetics in the acute phase are driven predominantly by ischemia–reperfusion (I/R) injury rather than baseline disease burden alone. Mechanistically, the restoration of blood flow during PCI triggers a surge in oxidative stress and inflammatory signaling, leading to the rapid upregulation of Galectin-3 expression in activated macrophages and damaged cardiomyocytes [66]. Consequently, elevated post-PCI Galectin-3 may serve as a surrogate marker for the severity of this procedural inflammatory response, reflecting the degree of reperfusion injury and early maladaptive remodeling that pre-PCI levels fail to capture [67]. This highlights the critical importance of sampling timing, as the biomarker’s prognostic value appears to be “unmasked” only after the procedural insult. This interpretation is further supported by heterogeneity analysis demonstrating that variability in post-PCI Galectin-3 was significantly driven by disease spectrum, suggesting that the intensity of this inflammatory response varies predictably across different ACS subtypes.

Traditionally, risk stratification in ACS patients is performed using established clinical scoring systems, such as the Global Registry of Acute Coronary Events (GRACE) and the Thrombolysis in Myocardial Infarction (TIMI) scores [68,69]. These tools integrate baseline hemodynamic and demographic data to predict ischemic and mortality risks [2]. However, the biomarkers evaluated in this meta-analysis—particularly sST2 and GDF-15—provide incremental value by reflecting distinct pathophysiological pathways, such as myocardial fibrosis and systemic inflammatory stress, which are not fully captured by these conventional scoring systems. Several included studies have demonstrated that integrating these biomarkers into standard clinical models significantly enhances their predictive accuracy for long-term adverse events in ACSPCI patients [20,45]. This suggests that a multimarker approach, combining clinical scores with novel inflammatory–oxidative stress markers, may offer a more nuanced and precise risk stratification compared to existing tools alone.

A key strength of this meta-analysis is the rigorous assessment of the certainty of evidence using the GRADE framework. Despite the inherent limitations of observational designs, the majority of our findings—particularly for sLOX-1, GDF-15, and OPG—were graded as high-certainty. This assessment was largely driven by the large and very large effect sizes observed, which suggest that the prognostic signals of these biomarkers are robust and clinically relevant, overshadowing concerns regarding statistical heterogeneity. However, prognostic value for post-PCI sST2 and pre-PCI Galectin-3 should be interpreted with caution, as their certainty of evidence was graded as low. This was primarily due to imprecision, indicated by wide confidence intervals crossing the null value, suggesting that current data are insufficient to draw definitive prognostic conclusions for these specific settings.

Several limitations warrant consideration. First, the limited number of studies for certain markers, such as sLOX-1 and OPG, restricted the capacity for comprehensive subgroup analyses. Second, despite stratification by clinical spectrum and geography, considerable residual heterogeneity remained, suggesting that unmeasured factors like assay sensitivity or laboratory protocols contributed to the variance. Third, to maximize data inclusion, the conversion of medians and IQRs to means and SDs may have introduced bias due to the typically skewed nature of biomarker data. Finally, the included studies exhibited substantial methodological variability, including heterogeneous clinical outcome definitions, a lack of standardized cut-off values for “high” biomarker levels, and inconsistent covariate adjustments in multivariate HR models. These inconsistencies collectively influence the pooled risk estimates and complicate the establishment of universal clinical thresholds for risk stratification.

Despite these limitations, this meta-analysis provides a comprehensive synthesis of multiple pathways in the ACS-PCI context. The results suggest that a multimarker approach, incorporating both baseline inflammatory status and post-procedural stress responses, has the potential to enhance the accuracy of risk stratification. Future research should focus on standardized sampling timepoints and head-to-head comparisons of these biomarkers to establish clinically relevant thresholds to guide personalized intervention strategies.

## 5. Conclusions

This systematic review and meta-analysis indicates that certain inflammatory and oxidative stress biomarkers show significant prognostic associations for predicting major adverse cardiovascular events in patients with ACS undergoing PCI. Elevated pre-procedural levels of sST2, GDF-15, sLOX-1, and OPG are significantly associated with a higher risk of MACE, with sST2 and GDF-15 emerging as the most reliable prognostic indicators across different timeframes.

This study further highlights the critical importance of sampling timing. While pre-procedural measurements reflect the patient’s baseline risk, systemic inflammation state, and plaque instability, post-procedural levels may be influenced by the acute inflammatory response induced by the PCI procedure itself. These findings suggest that pre-procedural biomarker assessment might provide a more stable signal for risk stratification. However, given the observational nature of the included studies, these results should be interpreted as prognostic associations rather than direct causality. Future research should focus on establishing standardized cut-off values and exploring the efficacy of a multimarker approach to enhance the precision of long-term prognostic monitoring among high-risk populations.

## Figures and Tables

**Table 2:** Summary of subgroup analysis.

Marker	Sampling Timepoint	Subgroup Analysis Variable	Number of Studies per Subgroup	Measurement Effect	95% Cl	*p*-Value
sST2	Pre-PCI	Follow-up (short-term)	2	SMD = 1.21	0.42–2.01	0.003 *
		Follow-up (medium-term)	6	SMD = 0.65	0.22–1.07	0.003 *
		Follow-up (long-term)	2	SMD = 0.62	(−0.14)–1.38	0.11
		Clinical outcome (MACE)	5	SMD = 0.77	0.25–1.30	0.004 *
		Clinical outcome (MACCE)	2	SMD = 0.63	(−0.20)–1.47	0.14
		Clinical outcome (CVD)	2	SMD = 0.62	(−0.23)–1.48	0.15
		Clinical outcome (mortality/HF)	1	SMD = 1.20	(−0.14)–2.54	0.08
		Continent (Asian)	7	SMD = 0.80	0.39–1.21	0.0001 *
		Continent (European)	3	SMD = 0.62	(−0.02)–1.25	0.06
		Disease (STEMI)	8	SMD = 0.64	0.31–0.97	0.0001 *
		Disease (NSTEMI)	1	SMD = 0.60	(−0.28)–1.48	0.18
		Disease (AMI)	1	SMD = 1.65	0.74–2.56	0.0004 *
		Follow-up (medium-term)	7	HR = 3.18	2.26–4.49	<0.0001 *
		Follow-up (long-term)	1	HR = 3.88	1.14–13.20	0.03 *
		Clinical outcome (MACE)	4	HR = 2.66	1.66–4.25	<0.0001 *
		Clinical outcome (MACCE)	3	HR = 4.07	2.36–7.02	<0.0001 *
		Clinical outcome (CVD)	1	HR = 3.88	1.11–13.51	0.03 *
		Disease (STEMI)	5	HR = 2.88	1.98–4.17	<0.0001 *
		Disease (NSTEMI)	1	HR = 3.35	1.90–5.92	<0.0001 *
		Disease (NSTEMI, UA)	1	HR = 10.22	4.06–25.74	<0.0001 *
		Disease (AMI)	1	HR = 2.26	1.98–4.17	0.02 *
		Continent (Asian)	7	HR = 3.18	2.26–4.49	<0.0001 *
		Continent (European)	1	HR = 3.88	1.14–13.20	0.03 *
	Post-PCI	Follow-up (short-term)	2	SMD = 0.11	(−0.76)–0.97	0.81
		Follow-up (medium-term)	2	SMD = 0.45	(−0.39)–(1.29)	0.30
		Clinical outcome (MACE)	3	SMD = 0.1581	(−0.48)–0.80	0.63
		Clinical outcome (all-cause mortality)	1	SMD = 0.6971	(−0.49)–1.88	0.25
		Disease (STEMI)	3	SMD = 0.16	(−0.48)–0.80	0.63
		Disease (ACS with cardiogenic shock)	1	SMD = 0.70	(−0.49)–1.88	0.25
		Continent (European)	1	SMD = 0.74	(−0.14)–1.62	0.10
		Continent (Asian)	2	SMD = −0.11	(−0.70)–0.48	0.71
		Continent (multicenter)	1	SMD = 0.70	(−0.24)–1.64	0.15
GDF-15	Pre-PCI	Follow-up (short-term)	2	HR = 2.74	1.48–5.07	0.001 *
		Follow-up (long-term)	3	HR = 3.28	1.94–5.55	<0.0001 *
		Clinical outcome (MACE)	2	HR = 2.82	1.79–4.43	<0.0001 *
		Clinical outcome (CVD)	1	HR = 2.27	1.29–4.00	0.005 *
		Clinical outcome (all-cause mortality)	1	HR = 2.45	1.06–5.66	0.04 *
		Clinical outcome (all-cause mortality, HF hospitalization)	1	HR = 6.30	2.94–13.49	<0.0001 *
		Continent (European)	3	HR = 3.31	1.22–10.40	<0.0001 *
		Continent (Asian)	1	HR = 3.56	1.82–6.01	0.02 *
		Continent (multicenter)	1	HR = 2.27	0.89–5.81	0.09
		Disease (ACS)	1	HR = 2.48	1.41–4.35	0.0015 *
		Disease (AMI)	2	HR = 4.74	2.76–8.14	<0.0001 *
		Disease (STEMI)	2	HR = 2.33	1.46–3.72	0.0004 *
H-FABP	Pre-PCI	Follow-up (short-term)	1	SMD = 0.74	0.10–1.38	0.02 *
		Follow-up (long-term)	2	SMD = 0.64	0.16–1.11	0.01 *
		Clinical outcome (CVD)	2	SMD = 0.64	0.16–1.11	0.01 *
		Clinical outcome (CVD, non-fatal AMI)	1	SMD = 0.74	0.10–1.38	0.02 *
		Continent (European)	1	SMD = 0.90	0.337–1.43	0.0009 *
		Continent (Asian)	2	SMD = 0.58	0.26–0.90	0.0004 *
		Disease (STEMI)	2	SMD = 0.64	0.16–1.11	0.01 *
		Disease (ACS)	1	SMD = 0.74	0.10–1.38	0.02 *
Galectin-3	Post-PCI	Continent (European)	2	SMD = 1.19	0.41–1.96	0.003 *
		Continent (American)	1	SMD = 1.09	(−0.09)–2.26	0.07
		Disease (STEMI)	2	SMD = 1.37	0.87–1.88	<0.0001 *
		Disease (AMI)	1	SMD = 0.81	0.24–1.38	0.005 *

* indicates statistical significance at *p* < 0.05.

**Table 3:** GRADE framework.

Outcome	GRADE Domains						Pooled Effect	Reasoning	Final GRADE Decision	Overall Certainty
	Study Design	Risk of Bias	Inconsistency	Indirectness	Imprecision	Other				
Pre-PCI sLOX-1 (SMD unit)	Non-randomized study	Not serious	Not serious	Not serious	Not serious	None	SMD = 0.38 (0.19–0.57)	None	None	⨁⨁⨁⨁ High
Pre-PCI H-FABP (SMD unit)	Non-randomized study	Not serious	Not serious	Not serious	Not serious	None	SMD = 0.67 (0.39–0.94)	None	None	⨁⨁⨁⨁ High
Pre-PCI OPG (SMD unit)	Non-randomized study	Not serious	Serious	Not serious	Not serious	Very large effect	SMD = 1.42 (0.40–2.44)	Substantial heterogeneity (I^2^ = 58.79%); very large effect size (SMD > 1.2)	Downgraded 1 level for inconsistency and upgraded 2 level for very large effect	⨁⨁⨁⨁ High
Pre-PCI sST2 (SMD unit)	Non-randomized study	Not serious	Serious	Not serious	Not serious	None	SMD = 0.75 (0.42–1.07)	Substantial heterogeneity (I^2^ = 87.37%)	Downgraded 1 level for inconsistency	⨁⨁⨁◯ Moderate
Pre-PCI GDF-15 (SMD unit)	Non-randomized study	Not serious	Serious	Not serious	Not serious	Very large effect	SMD = 1.55 (0.74–2.36)	Substantial heterogeneity (I^2^ = 87.40%); very large effect (SMD > 1.2)	Downgraded 1 level for inconsistency and upgraded 2 level for very large effect	⨁⨁⨁⨁ High
Pre-PCI Galectin-3 (SMD unit)	Non-randomized study	Not serious	Serious	Not serious	Serious	None	SMD = 1.58 (−0.26–3.43)	Substantial heterogeneity (I^2^ = 97.25%); crosses null value	Downgraded 2 level for inconsistency and imprecision	⨁⨁◯◯ Low
Post-PCI sST2 (SMD unit)	Non-randomized study	Not serious	Serious	Not serious	Serious	None	SMD = 0.28 (−0.25–0.81)	Substantial heterogeneity (I^2^ = 87.49%); crosses null value	Downgraded 2 level for inconsistency and imprecision	⨁⨁◯◯ Low
Post-PCI GDF-15 (SMD unit)	Non-randomized study	Not serious	Not serious	Not serious	Not serious	Large effect	SMD = 0.84 (0.52–1.15)	None	None	⨁⨁⨁⨁ High
Post-PCI Galectin-3 (SMD unit)	Non-randomized study	Not serious	Serious	Not serious	Not serious	Large effect	SMD = 1.15 (0.66–1.63)	Substantial heterogeneity (I^2^ = 61.78%); large effect (SMD > 0.8)	Downgraded 1 level for inconsistency and upgraded 1 level for large effect	⨁⨁⨁⨁ High
Pre-PCI sLOX-1 (HR unit)	Non-randomized study	Not serious	Not serious	Not serious	Not serious	Large effect	HR = 2.61 (1.41–4.85)	Large effect (HR > 2)	Upgraded 1 level for large effect	⨁⨁⨁⨁ High
Pre-PCI OPG (HR unit)	Non-randomized study	Not serious	Not serious	Not serious	Not serious	None	HR = 1.79 (1.09–2.92)	None	None	⨁⨁⨁⨁ High
Pre-PCI sST2 (HR unit)	Non-randomized study	Not serious	Not serious	Not serious	Not serious	Large effect	HR = 3.22 (2.36–4.40)	Large effect (HR > 2)	Upgraded 1 level for large effect	⨁⨁⨁⨁ High
Pre-PCI GDF-15 (HR unit)	Non-randomized study	Not serious	Not serious	Not serious	Not serious	Large effect	HR = 3.00 (2.12–4.25)	Large effect (HR > 2)	Upgraded 1 level for large effect	⨁⨁⨁⨁ High
Post-PCI sST2 (HR unit)	Non-randomized study	Not serious	Not serious	Not serious	Very serious	None	HR = 4.00 (0.93–17.15)	Crosses null value and wide confidence intervals	Downgraded 2 level for imprecision	⨁⨁◯◯ Low

⨁⨁⨁⨁ High certainty; ⨁⨁⨁◯ Moderate certainty; ⨁⨁◯◯ Low certainty; ⨁◯◯◯ Very low certainty.

## Data Availability

No new data were created or analyzed in this study. Data sharing is not applicable to this article.

## Supplementary Materials

The following supporting information can be downloaded at https://www.mdpi.com/article/10.3390/ijms27083389/s1.
