# Supplementary material for: The Prognostic Value of Pre-Procedural and Post-Procedural Inflammatory–Oxidative Stress Biomarkers in Acute Coronary Patients Undergoing Percutaneous Coronary Intervention: A Systematic Review and Meta-Analysis
Source: Int J Mol Sci. 2026 Apr 9;27(8):3389. doi: 10.3390/ijms27083389 (PMC13115952; doi:10.3390/ijms27083389)
Supplement: Supplementary file 1 [file ijms-27-03389-s001.zip › Table S2. CHARMS Checklist.pdf]

Table S2. CHARMS Checklist

CHARMS 2014 Relevant items to extract from individual studies in a systematic review of prediction models

Domain	Key items	Reported on page #
**SOURCE OF DATA**	Source of data (e.g., cohort, case-control, randomized trial participants, or registry data)	3-4
**PARTICIPANTS**	Participant eligibility and recruitment method (e.g., consecutive participants, location, number of centers, setting, inclusion and exclusion criteria)	3-4
	Participant description	6-13
	Details of treatments received, if relevant	3, 8-13
	Study dates	8-13
**OUTCOME(S) TO BE PREDICTED**	Definition and method for measurement of outcome	4
	Was the same outcome definition (and method for measurement) used in all patients?	4, 8-13
	Type of outcome (e.g., single or combined endpoints)	4
	Was the outcome assessed without knowledge of the candidate predictors (i.e., blinded)?	7
	Were candidate predictors part of the outcome (e.g., in panel or consensus diagnosis)?	4
	Time of outcome occurrence or summary of duration of follow-up	6-13
**CANDIDATE PREDICTORS (OR INDEX TESTS)**	Number and type of predictors (e.g., demographics, patient history, physical examination, additional testing, disease characteristics)	3, 8-13
	Definition and method for measurement of candidate predictors	4, 8-13
	Timing of predictor measurement (e.g., at patient presentation, at diagnosis, at treatment initiation)	3-4, 8-13
	Were predictors assessed blinded for outcome, and for each other (if relevant)?	7
	Handling of predictors in the modelling (e.g., continuous, linear, non-linear transformations or categorised)	5-6
**SAMPLE SIZE**	Number of participants and number of outcomes/events	6, 8-13
	Number of outcomes/events in relation to the number of candidate predictors (Events Per Variable)	Not applicable
	Number of participants with any missing value (include predictors and outcomes)	5
	Number of participants with missing data for each predictor	5

**MISSING DATA**	Handling of missing data (e.g., complete-case analysis, imputation, or other methods)	5-6
**MODEL DEVELOPMENT**	Modelling method (e.g., logistic, survival, neural network, or machine learning techniques)	5-6
	Modelling assumptions satisfied	5-6
	Method for selection of predictors **for inclusion** in multivariable modelling (e.g., all candidate predictors, pre-selection based on unadjusted association with the outcome)	4-5
	Method for selection of predictors **during multivariable modelling** (e.g., full model approach, backward or forward selection) and criteria used (e.g., p-value, Akaike Information Criterion)	Not applicable
	Shrinkage of predictor weights or regression coefficients (e.g., no shrinkage, uniform shrinkage, penalized estimation)	Not applicable
**MODEL PERFORMANCE**	Calibration (calibration plot, calibration slope, Hosmer-Lemeshow test) and Discrimination (C-statistic, D-statistic, log-rank) measures with confidence intervals	Not applicable
	Classification measures (e.g., sensitivity, specificity, predictive values, net reclassification improvement) and whether a-priori cut points were used	5-6, 8-13
**MODEL EVALUATIO N**	Method used for testing model performance: development dataset only (random split of data, resampling methods e.g. bootstrap or cross-validation, none) or separate external validation (e.g. temporal, geographical, different setting, different investigators)	Not applicable
	In case of poor validation, whether model was adjusted or updated (e.g., intercept recalibrated, predictor effects adjusted, or new predictors added)	Not applicable
**RESULTS**	Final and other multivariable models (e.g., basic, extended, simplified) presented, including predictor weights or regression coefficients, intercept, baseline survival, model performance measures (with standard errors or confidence intervals)	13-18
	Any alternative presentation of the final prediction models, e.g., sum score, nomogram, score chart, predictions for specific risk subgroups with performance	18-20
	Comparison of the distribution of predictors (including missing data) for development and validation datasets	Not applicable
**INTERPRETATIO N AND DISCUSSION**	Interpretation of presented models (confirmatory, i.e., model useful for practice versus exploratory, i.e., more research needed)	23-25
	Comparison with other studies, discussion of generalizability, strengths and limitations.	23-26
