# Supplementary material for: The Prognostic Value of Pre-Procedural and Post-Procedural Inflammatory–Oxidative Stress Biomarkers in Acute Coronary Patients Undergoing Percutaneous Coronary Intervention: A Systematic Review and Meta-Analysis
Source: Int J Mol Sci. 2026 Apr 9;27(8):3389. doi: 10.3390/ijms27083389 (PMC13115952; doi:10.3390/ijms27083389)
Supplement: Supplementary file 1 [file ijms-27-03389-s001.zip › Table S3. Search Strategy.pdf]

Table S3. Search Strategy

Database	Keywords
**Pubmed**	("soluble lectin-like oxidized low-density lipoprotein receptor-1" OR "sLOX-1" OR "soluble LOX-1" OR "sLOX1" OR "Heart-type fatty acid-binding protein" OR "H-FABP" OR "FABP3" OR "Osteoprotegerin" OR "OPG" OR "Soluble ST2" OR "sST2" OR "Growth Differentiation Factor-15" OR "GDF-15" OR "Galectin-3") AND ("acute coronary syndrome" OR "ACS" OR "myocardial infarction" OR "STEMI" OR "NSTEMI" OR "unstable angina") AND ("percutaneous coronary intervention" OR "PCI" OR "angioplasty" OR "stent") AND ("prognosis" OR "major adverse cardiac events" OR "MACE" OR "mortality" OR "death" OR "recurrence" OR "rehospitalization")
**Scopus**	("soluble lectin-like oxidized low-density lipoprotein receptor-1" OR "sLOX-1" OR "soluble LOX-1" OR "sLOX1" OR "Heart-type fatty acid-binding protein" OR "H-FABP" OR "FABP3" OR "Osteoprotegerin" OR "OPG" OR "Soluble ST2" OR "sST2" OR "Growth Differentiation Factor-15" OR "GDF-15" OR "Galectin-3") AND ("acute coronary syndrome" OR "ACS" OR "myocardial infarction" OR "STEMI" OR "NSTEMI" OR "unstable angina") AND ("percutaneous coronary intervention" OR "PCI" OR "angioplasty" OR "stent") AND ("prognosis" OR "major adverse cardiac events" OR "MACE" OR "mortality" OR "death" OR "recurrence" OR "rehospitalization")
**Web of Science**	("soluble lectin-like oxidized low-density lipoprotein receptor-1" OR "sLOX-1" OR "soluble LOX-1" OR "sLOX1" OR "Heart-type fatty acid-binding protein" OR "H-FABP" OR "FABP3" OR "Osteoprotegerin" OR "OPG" OR "Soluble ST2" OR "sST2" OR "Growth Differentiation Factor-15" OR "GDF-15" OR "Galectin-3") AND ("acute coronary syndrome" OR "ACS" OR "myocardial infarction" OR "STEMI" OR "NSTEMI" OR "unstable angina") AND ("percutaneous coronary intervention" OR "PCI" OR "angioplasty" OR "stent") AND ("prognosis" OR "major adverse cardiac events" OR "MACE" OR "mortality" OR "death" OR "recurrence" OR "rehospitalization")
**Proquest**	("soluble lectin-like oxidized low-density lipoprotein receptor-1" OR "sLOX-1" OR "soluble LOX-1" OR "sLOX1" OR "Heart-type fatty acid-binding protein" OR "H-FABP" OR "FABP3" OR "Osteoprotegerin" OR "OPG" OR "Soluble ST2" OR "sST2" OR "Growth Differentiation Factor-15" OR "GDF-15" OR "Galectin-3") AND ("acute coronary syndrome" OR "ACS" OR "myocardial infarction" OR "STEMI" OR "NSTEMI" OR "unstable angina") AND ("percutaneous coronary intervention" OR "PCI" OR "angioplasty" OR "stent") AND ("prognosis" OR "major adverse cardiac events" OR "MACE" OR "mortality" OR "death" OR "recurrence" OR "rehospitalization")
**ScienceDirect**	("Osteoprotegerin" OR "Soluble LOX-1" OR "Soluble ST2" OR "Growth Differentiation Factor-15" OR "Galectin-3" OR "heart-type fatty acid-binding protein") AND ("ACS" OR "STEMI" OR "NSTEMI" OR "Myocardial Infarction") AND ("PCI") AND ("Prognosis" OR "MACE" OR "mortality")
**Sage**	("soluble lectin-like oxidized low-density lipoprotein receptor-1" OR "sLOX-1" OR "soluble LOX-1" OR "sLOX1" OR "Heart-type fatty acid-binding protein" OR "H-FABP" OR "FABP3" OR "Osteoprotegerin" OR "OPG" OR "Soluble ST2" OR "sST2" OR "Growth Differentiation Factor-15" OR "GDF-15" OR "Galectin-3") AND ("acute coronary syndrome" OR "ACS" OR "myocardial infarction" OR "STEMI" OR "NSTEMI" OR "unstable angina") AND ("percutaneous coronary intervention" OR "PCI" OR "angioplasty" OR "stent") AND ("prognosis" OR "major adverse cardiac events" OR "MACE" OR "mortality" OR "death" OR "recurrence" OR "rehospitalization")
**Taylor & Francis**	("soluble lectin-like oxidized low-density lipoprotein receptor-1" OR "sLOX-1" OR "soluble LOX-1" OR "sLOX1" OR "Heart-type fatty acid-binding protein" OR "H-FABP" OR "FABP3" OR "Osteoprotegerin" OR "OPG" OR "Soluble ST2" OR "sST2" OR "Growth

	Differentiation Factor-15" OR "GDF-15" OR "Galectin-3") AND ("acute coronary syndrome" OR "ACS" OR "myocardial infarction" OR "STEMI" OR "NSTEMI" OR "unstable angina") AND ("percutaneous coronary intervention" OR "PCI" OR "angioplasty" OR "stent") AND ("prognosis" OR "major adverse cardiac events" OR "MACE" OR "mortality" OR "death" OR "recurrence" OR "rehospitalization")
**Springer**	("soluble lectin-like oxidized low-density lipoprotein receptor-1" OR "sLOX-1" OR "soluble LOX-1" OR "sLOX1" OR "Heart-type fatty acid-binding protein" OR "H-FABP" OR "FABP3" OR "Osteoprotegerin" OR "OPG" OR "Soluble ST2" OR "sST2" OR "Growth Differentiation Factor-15" OR "GDF-15" OR "Galectin-3") AND ("acute coronary syndrome" OR "ACS" OR "myocardial infarction" OR "STEMI" OR "NSTEMI" OR "unstable angina") AND ("percutaneous coronary intervention" OR "PCI" OR "angioplasty" OR "stent") AND ("prognosis" OR "major adverse cardiac events" OR "MACE" OR "mortality" OR "death" OR "recurrence" OR "rehospitalization")
**Medrxiv**	("Osteoprotegerin" OR "soluble LOX-1" OR "H-FABP" OR "soluble ST2" OR "GDF-15" OR "Galectin-3") AND ("Acute Coronary Syndrome" OR "STEMI" OR "NSTEMI" OR "Unstable Angina") AND ("Prognosis" OR "MACE")
**Bioxriv**	("Osteoprotegerin" OR "soluble LOX-1" OR "H-FABP" OR "soluble ST2" OR "GDF-15" OR "Galectin-3") AND ("Acute Coronary Syndrome" OR "STEMI" OR "NSTEMI" OR "Unstable Angina") AND ("Prognosis" OR "MACE")
