# Supplementary material for: The Prognostic Value of Pre-Procedural and Post-Procedural Inflammatory–Oxidative Stress Biomarkers in Acute Coronary Patients Undergoing Percutaneous Coronary Intervention: A Systematic Review and Meta-Analysis
Source: Int J Mol Sci. 2026 Apr 9;27(8):3389. doi: 10.3390/ijms27083389 (PMC13115952; doi:10.3390/ijms27083389)
Supplement: Supplementary file 1 [file ijms-27-03389-s001.zip › Table S4. Exclusion Studies.pdf]

Table S4. Exclusion Study

Author (Year)	Title	Reason for Exclusion
Viswanathan et al. (2010)	Heart-Type Fatty Acid-Binding Protein Predicts Long-Term Mortality and Re-Infarction in Consecutive Patients With Suspected Acute Coronary Syndrome Who Are Troponin-Negative	Different population (Cohort consisted of suspected ACS patients, not confirmed ACS undergoing PCI)
Jones et al. (2025)	Risk Stratification of Acute Chest Pain in Patients With High-Sensitivity Troponin T Below the 99th Percentile: A Long-Term Cohort Study Assessing the Incremental Value of Necrosis and Non-necrosis Biomarkers to Clinical Risk Scores	Different population (Evaluated patients with suspected ACS where acute myocardial infarction was ultimately ruled out)
Mustafa et al. (2025)	Diagnostic and Prognostic Evaluation of Novel Biomarkers Compared to ESC 0/1 h and 0/3 h Algorithms in Patients with Suspected Non-ST-Elevation Myocardial Infarction	Different population (Evaluated patients with suspected NSTEMI in the emergency department, not a specific post-PCI cohort)
Kilcullen et al. (2007)	Heart-Type Fatty Acid-Binding Protein Predicts Long-Term Mortality After Acute Coronary Syndrome and Identifies High-Risk Patients Across the Range of Troponin Values	Different population (General ACS registry cohort, not exclusively limited to patients undergoing PCI)
O'Donoghue et al. (2006)	Prognostic Utility of Heart-Type Fatty Acid Binding Protein in Patients With Acute Coronary Syndromes	Different population (General ACS clinical trial cohort, not specifically focused on PCI-treated patients)
Reiter et al. (2013)	Heart-type fatty acid-binding protein in the early diagnosis of acute myocardial infarction	Different outcome (Primary focus was on early diagnostic accuracy, not long-term prognostic risk stratification)
Matsumoto et al. (2013)	Elevated Serum Heart-Type Fatty Acid-Binding Protein in the Convalescent Stage Predicts Long-Term Outcome in Patients Surviving Acute Myocardial Infarction	Different method (Biomarker sampling was performed at the convalescent stage, not at the pre-procedural or acute post-procedural phase)
Suzuki et al. (2005)	Prognostic Value of a Qualitative Test for Heart-Type Fatty Acid-Binding Protein in Patients With Acute Coronary Syndrome	Different method (Utilized a qualitative point-of-care test rather than a quantitative concentration assay)
Shi et al. (2025)	Protective effects of combined eptifibatide and ticagrelor in patients with unstable angina undergoing percutaneous coronary intervention: a single-center experience	Different method (Intervention efficacy study where H-FABP was evaluated as a therapeutic outcome marker, not a baseline prognostic predictor)
Adlbrecht et al. (2010)	Long-term outcome after thrombectomy in acute myocardial infarction	Different method (Intervention comparison study where biomarker was measured as a secondary endpoint, not as the primary prognostic exposure)
Nakata et al. (2003)	Human Heart-Type Fatty Acid-Binding Protein as an Early Diagnostic and Prognostic Marker in Acute Coronary Syndrome	Different population (Cohort consisted of patients presenting to the emergency room with suspected ACS, not specifically confirmed ACS patients uniformly undergoing PCI)

Li et al. (2022)	Comparison of ticagrelor and clopidogrel on platelet function and prognosis in unstable angina	Different method (Intervention efficacy study comparing antiplatelet drugs where H-FABP was evaluated as an outcome marker of myocardial injury, not as the primary prognostic exposure)
Connolly et al. (2018)	Heart-type fatty acid-binding protein (H-FABP) and highly sensitive troponin T (hsTnT) as markers of myocardial injury and cardiovascular events in elective percutaneous coronary intervention (PCI)	Different population (Evaluated patients undergoing elective PCI, which typically indicates stable coronary artery disease, rather than Acute Coronary Syndrome)
Adlbrecht et al. (2010)	Long-term outcome after thrombectomy in acute myocardial infarction	Different method (Intervention comparison study where biomarker was measured as a secondary endpoint, not as the primary prognostic exposure)
Jadczyk et al. (2019)	Serum Concentrations of Osteogenesis/Osteolysis-Related Factors and Micro-RNA Expression in ST-Elevation Myocardial Infarction	Different outcome (Primary focus on molecular profiling and early remodeling, rather than long-term prognostic clinical events like MACE)
Cottin et al. (2021)	Association between Serum Osteoprotegerin Levels and Severity of Coronary Artery Disease in Patients with Acute Myocardial Infarction	Different outcome (Evaluated angiographic severity, such as SYNTAX score, rather than long-term prognostic MACE or mortality)
Shui et al. (2022)	Association of serum sclerostin and osteoprotegerin levels with the presence, severity and prognosis in patients with acute myocardial infarction	Different population (General AMI cohort, not specifically restricted to patients uniformly managed with PCI)
Siller-Matula et al. (2017)	Interdependence between osteoprotegerin and active von Willebrand factor in long-term cardiovascular mortality prediction in patients undergoing percutaneous coronary intervention	Different population (Cohort included patients with stable coronary artery disease undergoing PCI, not exclusively Acute Coronary Syndrome)
Pedersen et al. (2012)	Osteoprotegerin Predicts Long-Term Outcome in Patients with ST-Segment Elevation Myocardial Infarction Treated with Primary Percutaneous Coronary Intervention	No outcome values (Did not provide extractable hazard ratios or comparable continuous effect estimates based on elevated biomarker thresholds required for data synthesis)
Andersen et al. (2011)	Elevated serum osteoprotegerin levels measured early after acute ST-elevation myocardial infarction predict final infarct size	Different outcome (Primary focus was on evaluating final infarct size via SPECT imaging, rather than long-term prognostic clinical endpoints like MACE)
Shavadia et al. (2021)	Novel multi-marker proteomics in phenotypically matched patients with ST-segment myocardial infarction: association with clinical outcomes	Different method (Evaluated the prognostic value of the 24-hour relative temporal change in biomarker concentrations, rather than utilizing a fixed elevated concentration cut-off)
Fuernau et al. (2013)	Osteoprotegerin in ST-elevation myocardial infarction: Prognostic impact and association with	No outcome values (Did not provide extractable categorical adjusted hazard ratios for the primary prognostic

	markers of myocardial damage by magnetic resonance imaging	endpoints, as multivariable analysis only reported continuous variables)
Lindberg et al. (2014)	Osteoprotegerin Levels Change During STEMI and Reflect Cardiac Function	No outcome values (Study primarily evaluated left ventricular ejection fraction and did not report extractable hazard ratios for clinical outcomes due to an insufficient number of events during follow-up)
Tuñón et al. (2014)	Usefulness of a Combination of Monocyte Chemoattractant Protein-1, Galectin-3, and N-Terminal Probrain Natriuretic Peptide to Predict Cardiovascular Events in Patients With Coronary Artery Disease	Different population (Cohort consisted of patients with chronic coronary artery disease, rather than an acute ACS population specifically undergoing PCI)
Turan & Demir (2020)	The relation of endocan and galectin-3 with ST-segment resolution in patients with ST-segment elevation myocardial infarction	Different outcome (Evaluated acute electrocardiographic ST-segment resolution as the primary endpoint, not long-term prognostic clinical events like MACE)
Karagiannidis et al. (2021)	Correlation of the severity of coronary artery disease with patients' metabolic profile- rationale, design and baseline patient characteristics of the CorLipid trial	Different method (Study protocol and design rationale paper, not a completed observational study with reported long-term outcomes)
Kovalskaya et al. (2022)	Effect of combined lipid-lowering therapy on atherosclerotic plaque vulnerability in patients with acute coronary syndrome (Combi-LLT ACS): randomized trial protocol	Different method (Study protocol for an interventional randomized controlled trial, not a completed observational prognostic study)
Cheng et al. (2022)	Circulating Galectin-3 in Patients with Cardiogenic Shock Complicating Acute Myocardial Infarction Treated with Mild Hypothermia: A Biomarker Sub-Study of the SHOCK-COOL Trial	Different population (Sub-study restricted to patients with cardiogenic shock treated with mild hypothermia, which introduces significant intervention bias not representative of the general ACS-PCI population)
Salib et al. (2023)	Levels of Procollagen Type I C-Terminal Pro-Peptide and Galectin-3, Arterial Stiffness Measured By Pulse Wave Velocity, and Cardiovascular Morbidity and Mortality in 44 Patients 2 Years After Kidney Transplantation	Different population (Cohort consisted of kidney transplant recipients, not ACS patients undergoing PCI)
Mayyas (2023)	Evaluating Plasma Galectin-3 Levels in Patients With an Increased Risk of Atherosclerotic Cardiovascular Disease Who Underwent Coronary Artery Revascularization	Different population (Included patients with stable angina)
Jones et al. (2025)	Risk Stratification of Acute Chest Pain in Patients With High-Sensitivity Troponin T Below the 99th Percentile: A Long-Term Cohort Study Assessing the Incremental Value of Necrosis and Non-necrosis Biomarkers to Clinical Risk Scores	Different population (Evaluated patients with suspected acute coronary syndrome where acute myocardial infarction was ultimately ruled out)

Maiolino et al. (2015)	Galectin-3 Predicts Long-Term Cardiovascular Death in High-Risk Patients With Coronary Artery Disease	Different population (General high-risk CAD cohort undergoing coronary angiography, not specifically restricted to an ACS cohort treated with PCI)
Wang et al. (2025)	Serum galectin-3 level increase correlates with poor prognosis in acute coronary syndrome	No outcome values (Did not report standard extractable categorical hazard ratios or comparable continuous effect estimates for data synthesis, as the study primarily utilized Classification and Regression Tree [CART] analysis)
Idzikowska et al. (2022)	The Prognostic Value of Cardiac Biomarkers in Patients with Acute Myocardial Infarction during and after Hospitalization	No outcome values (Did not report standard extractable categorical hazard ratios or comparable continuous effect estimates for data synthesis, as the study utilized logistic regression yielding Odds Ratios)
Szadkowska et al. (2013)	The association between galectin-3 and clinical parameters in patients with first acute myocardial infarction treated with primary percutaneous coronary angioplasty	Different outcome (Primary focus was on evaluating clinical parameters in the acute phase, such as new-onset atrial fibrillation and diuretic use, rather than long-term prognostic clinical events like MACE)
Milner et al. (2014)	Temporal expression of galectin-3 following myocardial infarction	Different outcome (Primary focus was on the temporal kinetics of the biomarker and its correlation with left ventricular function, rather than long-term prognostic clinical endpoints)
Obeid et al. (2020)	Prognostic role of plasma galectin-3 levels in acute coronary syndrome	Different population (Cohort included ACS patients undergoing either PCI or CABG, not exclusively restricted to patients uniformly managed with PCI)
Tsai et al. (2012)	Value and Level of Galectin-3 in Acute Myocardial Infarction Patients Undergoing Primary Percutaneous Coronary Intervention	No outcome values (Did not report standard extractable time-to-event hazard ratios required for data synthesis; the study reported odds ratios for a 30-day composite outcome)
Asleh et al. (2019)*	Galectin-3 Levels and Outcomes After Myocardial Infarction: A Population-Based Study	Different population (Evaluated a population-based general incident myocardial infarction cohort, rather than a specific ACS cohort uniformly treated with PCI)
Hongisto et al. (2021)	Mortality risk prediction in elderly patients with cardiogenic shock: results from the CardShock study	Different population (Cohort was specifically restricted to patients with cardiogenic shock, which introduces significant selection and mortality bias compared to the general ACS-PCI population)
Li et al. (2021)	Prognostic value of soluble suppression of tumorigenesis-2 (sST2) for cardiovascular events	Different population (Cohort consisted of general coronary artery disease

	in coronary artery disease patients with and without diabetes mellitus	[CAD] patients, not exclusively an acute ACS population uniformly undergoing PCI)
Chen et al. (2023)*	Elevated Soluble Suppressor of Tumorigenicity 2 Predict Hospital Admissions Due to Major Adverse Cardiovascular Events (MACE)	Different population (Evaluated a general cardiovascular/heart failure cohort rather than a specific ACS cohort treated with PCI)
Zheng et al. (2025)	Serum sST2, IL-33, and Hcy Expression in Older Adults Patients with Myocardial Infarction and Their Predictive Value for MACE	Different population (Cohort restricted to elderly patients [≥65 years] and not explicitly limited to those uniformly managed with PCI)
Zhang et al. (2025)	Association between soluble suppression of tumorigenicity 2 and risk and severity of coronary artery disease: a case control study	Different outcome (Primary focus was on diagnostic risk and angiographic severity using the Gensini score, rather than long-term prognostic clinical events)
Huang et al. (2025)	Short-term predictive value of sST2 in patients with STEMI following primary PCI: a prospective observational study	No outcome values (Did not report standard extractable adjusted hazard ratios [aHR] for data synthesis; only reported categorical MACE incidence rates without time-to-event multivariable adjustment)
Bai et al. (2020)	Predictive value of soluble suppression of tumourigenicity 2 on myocardial reperfusion	Different outcome (Primary endpoint was angiographic myocardial reperfusion / TIMI flow grading, rather than long-term prognostic clinical events like MACE)
Somuncu et al. (2019)	The Elevated Soluble ST2 Predicts No-Reflow Phenomenon in ST-Elevation Myocardial Infarction Undergoing Primary Percutaneous Coronary Intervention	Different outcome (Primary endpoint was the angiographic no-reflow phenomenon, not long-term prognostic clinical events)
Søndergaard et al. (2022)	Soluble ST2 in plasma is associated with post-procedural no-or-slow reflow after primary percutaneous coronary intervention in ST-elevation myocardial infarction	Different outcome (Primary endpoint was post-procedural angiographic no-or-slow reflow, not long-term prognostic clinical events)
Witkowska et al. (2023)	Interleukin-33/sST2: Dynamic assessment in patients with acute coronary syndrome	Different outcome (Primary focus was on dynamic temporal changes of the biomarker rather than providing extractable adjusted hazard ratios for long-term prognostic clinical events)
Jenkins et al. (2017)	Prognostic Value of Soluble ST2 after Myocardial Infarction: A Community Perspective	Different population (Evaluated a general community-based myocardial infarction cohort, not exclusively restricted to an ACS cohort uniformly undergoing PCI)
Kim et al. (2021)	Lack of prognostic significance for major adverse cardiac events of soluble suppression of	No outcome values (Did not report standard extractable categorical hazard

	tumorigenicity 2 levels in patients with ST-segment elevation myocardial infarction	ratios or comparable continuous effect estimates required for data synthesis)
Jacobs et al. (2018)	The Association Between Novel Biomarkers and 1-Year Readmission or Mortality After Cardiac Surgery	Different population (Cohort consisted of patients undergoing cardiac surgery/CABG, rather than ACS patients undergoing PCI)
Jha et al. (2018)	Prognostic role of soluble ST2 in Acute Coronary Syndrome with Diabetes	Different population (Mixed cohort where only a sub-proportion of the patients [42.6%] underwent PCI, rather than an exclusive post-PCI cohort)
Pfetsch et al. (2017)	Increased Plasma Concentrations of Soluble ST2 Independently Predict Mortality but not Cardiovascular Events in Stable Coronary Heart Disease Patients	Different population (Evaluated patients with stable coronary heart disease, not an acute coronary syndrome cohort)
Minamisawa et al. (2016)	Comparison of Inflammatory Biomarkers in Outpatients With Prior Myocardial Infarction	Different method (Biomarker sampling was performed in the convalescent stage [one month after AMI in an outpatient setting], not at the pre-procedural or acute post-procedural phase)
Kohli et al. (2012)	Role of ST2 in Non-ST-Elevation Acute Coronary Syndrome in the MERLIN-TIMI 36 Trial	Different population (Evaluated a general clinical trial cohort of NSTE-ACS patients with mixed management strategies, not specifically restricted to those uniformly undergoing PCI)
Dhillon et al. (2011)	Interleukin 33 and ST2 in non-ST-elevation myocardial infarction: Comparison with Global Registry of Acute Coronary Events Risk Scoring and NT-proBNP	Different population (General NSTEMI cohort managed with mixed revascularization/medical strategies, not exclusively restricted to patients uniformly treated with PCI)
Andrup et al. (2024)	Novel cardiac extracellular matrix biomarkers in STEMI: Associations with ischemic injury and long-term mortality	No outcome values (Did not report standard extractable categorical adjusted hazard ratios based on elevated biomarker thresholds; analysis primarily relied on continuous variables)
Wallentin et al. (2016)	Early invasive versus non-invasive treatment in patients with non-ST-elevation acute coronary syndrome (FRISC-II): 15 year follow-up of a prospective, randomised, multicentre study	Different population (General NSTE-ACS clinical trial cohort managed with mixed early invasive and non-invasive strategies, not exclusively restricted to patients uniformly treated with PCI)
Rueda et al. (2019)	Acute-phase dynamics and prognostic value of growth differentiation factor-15 in ST-elevation myocardial infarction	No outcome values (Did not provide standard extractable adjusted hazard ratios [aHR] for the specific prognostic endpoints required for data synthesis)
Mayer et al. (2021)	The coincidence of low vitamin K status and high expression of growth differentiation factor 15 may indicate increased mortality risk in stable coronary heart disease patients	Different population (Evaluated patients with stable coronary heart disease, not an acute coronary syndrome cohort)

Batra et al. (2022)	Biomarker-Based Prediction of Recurrent Ischemic Events in Patients With Acute Coronary Syndromes	Different population (Analyzed clinical trial cohorts [PLATO and TRACER] utilizing mixed medical and invasive management strategies, not exclusively restricted to PCI)
Kożuch et al. (2023)	Growth Differentiation Factor 15 as a Predictor of the No-Reflow Phenomenon in Patients with ST-Segment Elevation Myocardial Infarction	Different outcome (Primary endpoint was the angiographic no-reflow phenomenon, rather than long-term clinical prognostic events like MACE)
Jones et al. (2025)	Risk Stratification of Acute Chest Pain in Patients With High-Sensitivity Troponin T Below the 99th Percentile: A Long-Term Cohort Study Assessing the Incremental Value of Necrosis and Non-necrosis Biomarkers to Clinical Risk Scores	Different population (Evaluated patients with suspected ACS where acute myocardial infarction was ultimately ruled out)
Dogdu (2020)	Assessment of Growth Differentiation Factor 15 Levels on Coronary Flow in Patients with STEMI Undergoing Primary PCI	Different outcome (Primary endpoint was angiographic coronary flow/TIMI grade success, not long-term clinical prognostic events)
Shavadia et al. (2021)	Novel multi-marker proteomics in phenotypically matched patients with ST-segment myocardial infarction: association with clinical outcomes	Different method (Evaluated the prognostic value of the 24-hour relative temporal change in biomarker concentrations, rather than utilizing a fixed elevated concentration cut-off at baseline)
Farhan et al. (2016)	Determinants of growth differentiation factor 15 in patients with stable and acute coronary artery disease. A prospective observational study	Different population (Cohort consisted of a mixture of stable coronary artery disease and ACS patients, not purely an ACS-PCI cohort)
Tentzeris et al. (2017)	Usefulness of Elevated Levels of Growth Differentiation Factor-15 to Classify Patients With Acute Coronary Syndrome Having Percutaneous Coronary Intervention Who Would Benefit from High-Dose Statin Therapy	Different method (Primary focus was evaluating the interaction between biomarker levels and statin therapy efficacy, rather than the independent long-term prognostic value of the biomarker itself)
Mo et al. (2022)	Serum GDF-15 Predicts In-Hospital Mortality and Arrhythmic Risks in Patients With Acute Myocardial Infarction	Different outcome (Evaluated short-term/in-hospital mortality and arrhythmic events rather than long-term prognostic clinical endpoints like post-discharge MACE)
Eggers et al. (2009)	Growth-Differentiation Factor-15 for Long-Term Risk Prediction in Patients Stabilized After an Episode of Non-ST-Segment-Elevation Acute Coronary Syndrome	Different population (FRISC-II clinical trial cohort managed with mixed early invasive and non-invasive strategies, not exclusively restricted to patients uniformly treated with PCI)
Kempf et al. (2009)	Growth-Differentiation Factor-15 for Risk Stratification in Patients With Stable and Unstable Coronary Heart Disease: Results From the AtheroGene Study	Different population (Cohort included a mixture of patients with stable angina pectoris and acute coronary syndrome)

Bonaca et al. (2011)	Growth Differentiation Factor-15 and Risk of Recurrent Events in Patients Stabilized After Acute Coronary Syndrome: Observations From PROVE IT-TIMI 22	Different population (Clinical trial cohort of stabilized patients post-ACS evaluated for statin therapy, utilizing mixed revascularization/medical strategies)
Li et al. (2020)	Growth differentiation factor-15 is associated with cardiovascular outcomes in patients with coronary artery disease	Different population (Evaluated a general coronary artery disease [CAD] cohort, not specifically restricted to an acute ACS population uniformly undergoing PCI)
Wollert et al. (2007)	Prognostic Value of Growth-Differentiation Factor-15 in Patients With Non-ST-Elevation Acute Coronary Syndrome	Different population (GUSTO-IV clinical trial cohort managed with mixed medical or interventional strategies, not exclusively an acute PCI cohort)
Kempf et al. (2007)	Growth-differentiation factor-15 improves risk stratification in ST-segment elevation myocardial infarction	Different population (Cohort consisted of STEMI patients receiving fibrinolytic therapy, rather than primary PCI)
Damman et al. (2014)	Growth-differentiation factor 15 for long-term prognostication in patients with non-ST-elevation acute coronary syndrome: An Invasive versus Conservative Treatment in Unstable coronary Syndromes (ICTUS) substudy	Different population (ICTUS clinical trial cohort comparing invasive versus conservative treatment strategies, not a uniform PCI cohort)
Hagström et al. (2015)	Growth differentiation factor-15 level predicts major bleeding and cardiovascular events in patients with acute coronary syndromes: results from the PLATO study	Different population (PLATO clinical trial cohort utilizing mixed medical management, CABG, and PCI strategies, not exclusively restricted to PCI)
Lindholm et al. (2017)	Biomarkers and Coronary Lesions Predict Outcomes after Revascularization in Non-ST-Elevation Acute Coronary Syndrome	Different population (Sub-study of the PLATO clinical trial cohort, which utilized mixed management and revascularization strategies [including medical management and CABG], rather than an exclusive cohort of ACS patients uniformly treated with PCI)
Schiopu et al. (2025)	Circulating soluble LOX-1 and patient prognosis after an acute coronary syndrome	Different population (General ACS cohort, not exclusively restricted to patients uniformly managed with PCI)
Zhao et al. (2019)	Higher serum lectin-like oxidized low-density lipoprotein receptor-1 in patients with stable coronary artery disease is associated with major adverse cardiovascular events: A multicentre pilot study	Different population (Evaluated patients with stable coronary artery disease, not an acute coronary syndrome cohort)
Mashayekhi et al. (2018)	Prognostic value of sLOX-1 level in acute coronary syndromes based on thrombolysis in myocardial infarction risk score and clinical outcome	No outcome values (Did not report standard extractable multivariable-adjusted hazard ratios for independent prognostic evaluation required for data synthesis)

Kobayashi et al. (2013)	Soluble lectin-like oxidized LDL receptor-1 (sLOX-1) as a valuable diagnostic marker for rupture of thin-cap fibroatheroma: Verification by optical coherence tomography	Different outcome (Primary endpoint was morphological plaque rupture evaluated by intracoronary imaging, not long-term prognostic clinical events)
Jiao et al. (2015)	Efficacy and Safety of Loading-Dose Rosuvastatin Therapy in Elderly Patients with Acute Coronary Syndromes Undergoing Elective Percutaneous Coronary Intervention	Different method (Intervention efficacy study evaluating statin therapy, not an observational prognostic study of baseline biomarker levels)
Kook et al. (2020)	Identification of plaque ruptures using a novel discriminative model comprising biomarkers in patients with acute coronary syndrome	Different outcome (Primary focus was the acute diagnosis of plaque rupture, not long-term prognostic clinical endpoints like MACE)
Stankova et al. (2019)	Serum Levels of Carbamylated LDL and Soluble Lectin-Like Oxidized Low-Density Lipoprotein Receptor-1 Are Associated with Coronary Artery Disease in Patients with Metabolic Syndrome	Different population (Cohort consisted of patients with metabolic syndrome, not specifically an acute ACS cohort uniformly treated with PCI)
Kott et al. (2023)	Serum Soluble Lectin-like Oxidized Low-Density Lipoprotein Receptor-1 (sLOX-1) Is Associated with Atherosclerosis Severity in Coronary Artery Disease	Different population (Evaluated general CAD patients for baseline atherosclerosis severity, not an acute ACS-PCI cohort)
Caglar et al. (2016)	Association between soluble lectin-like oxidized low-density lipoprotein receptor 1 levels and coronary slow flow phenomenon	Different outcome (Primary endpoint was the angiographic coronary slow flow phenomenon, rather than long-term clinical prognostic events like MACE)
Osman et al. (2022)	Serum soluble lectin-like oxidized low-density lipoprotein receptor-I as a diagnostic marker for acute ST-elevation myocardial infarction	Different outcome (Primary focus was on acute diagnostic accuracy, not long-term prognostic risk stratification)
Kume et al. (2010)	Soluble Lectin-Like Oxidized Low-Density Lipoprotein Receptor-1 Predicts Prognosis After Acute Coronary Syndrome - A Pilot Study	No outcome values (Did not report standard extractable multivariable-adjusted hazard ratios required for quantitative data synthesis)
