# Supplementary material for: The Prognostic Value of Pre-Procedural and Post-Procedural Inflammatory–Oxidative Stress Biomarkers in Acute Coronary Patients Undergoing Percutaneous Coronary Intervention: A Systematic Review and Meta-Analysis
Source: Int J Mol Sci. 2026 Apr 9;27(8):3389. doi: 10.3390/ijms27083389 (PMC13115952; doi:10.3390/ijms27083389)
Supplement: Supplementary file 1 [file ijms-27-03389-s001.zip › Table S5. Results of quality assessment of cohort studies based on the Newcastle-Ottawa Scale..pdf]

**Table S5.** Results of quality assessment of cohort studies based on the Newcastle-Ottawa Scale.

| First author (year) | Selection | | | | Comparability | Exposure | | | Study quality | |
|---|---|---|---|---|---|---|---|---|---|---|
| | Representativeness of the exposed cohort | Selection of the non-exposed cohort | Ascertainment of exposure | Demonstration that outcome of interest was not present at start of study | Comparability of cohorts on the basis of the design or analysis | Assessment of outcome | Enough follow-up time length for outcome to occur | Adequacy of follow-up of cohorts | Total score | Judgment |
| Kraler (2022)[24] | * | * | * | * | ** | * | * | * | 9 | High |
| Zhao (2019)[22] | * | * | * | * | * | * | * | 0 | 7 | High |
| Higuma (2015)[21] | * | * | * | * | * | * | * | 0 | 7 | High |
| Kumar (2021)[23] | 0 | * | * | * | 0 | * | * | * | 6 | Moderate |
| Kopp (2025)[17] | * | * | * | * | ** | * | * | * | 9 | High |
| Ishii (2005)[25] | 0 | * | * | * | * | * | * | 0 | 6 | Moderate |
| Erdal (2023)[26] | * | * | * | * | * | * | * | 0 | 7 | High |
| Bjerre (2014)[29] | * | * | * | * | ** | * | * | * | 9 | High |
| Hyseni (2013)[28] | * | * | * | * | ** | * | * | * | 9 | High |
| Canga (2012)[27] | * | * | * | * | * | * | 0 | * | 8 | High |

| | | | | | | | | | | |
|---|---|---|---|---|---|---|---|---|---|---|
| Fuernau (2014)[18] | 0 | * | * | * | ** | * | * | * | 8 | High |
| Lindberg (2014)[30] | 0 | * | * | * | ** | * | * | * | 8 | High |
| Huang (2018)[33] | * | * | * | * | 0 | * | * | * | 7 | High |
| Liu (2019)[34] | * | * | * | * | ** | * | * | * | 9 | High |
| Yu (2017)[32] | * | * | * | * | * | * | * | * | 8 | High |
| Wang (2017)[31] | * | * | * | * | * | * | * | * | 8 | High |
| Tyminska (2019)[19] | * | * | * | * | ** | * | * | 0 | 8 | High |
| Somuncu (2020)[35] | * | * | * | * | ** | * | * | * | 9 | High |
| Zagidullin (2020)[36] | * | * | * | * | ** | * | * | * | 9 | High |
| Che (2025)[40] | * | * | * | * | ** | * | * | * | 9 | High |
| Zhang (2021)[37] | * | * | * | * | * | * | * | * | 8 | High |
| Mechtouff (2022)[39] | * | * | * | * | ** | * | * | * | 9 | High |
| Liu (2022)[38] | * | * | * | * | ** | * | * | * | 9 | High |
| Xu (2025)[42] | * | * | * | * | ** | * | * | * | 9 | High |
| Lam (2025)[41] | * | * | * | * | 0 | * | * | * | 7 | High |

| | | | | | | | | | | |
|---|---|---|---|---|---|---|---|---|---|---|
| Yin (2025)[43] | * | * | * | * | ** | * | * | * | 9 | High |
| Hongisto (2021)[20] | 0 | * | * | * | * | * | * | * | 7 | High |
| Bodde (2019)[47] | * | * | * | * | ** | * | * | * | 9 | High |
| Velders (2015)[45] | * | * | * | * | ** | * | * | * | 9 | High |
| Peiro (2019)[48] | 0 | * | * | * | ** | * | * | 0 | 7 | High |
| Eitel (2011)[44] | * | * | * | * | ** | * | * | * | 9 | High |
| Sun (2018)[46] | * | * | * | * | ** | * | 0 | 0 | 7 | High |
| Peiro (2025)[50] | * | * | * | * | ** | * | * | 0 | 8 | High |
| Gurgoze (2023)[49] | * | * | * | * | ** | * | * | * | 9 | High |
| Di Tano (2017)[52] | * | * | * | * | ** | * | * | * | 9 | High |
| Gagno (2019)[54] | * | * | * | * | ** | * | * | * | 9 | High |
| Choi (2025)[56] | 0 | * | * | * | ** | * | * | * | 8 | High |
| Lisowska (2016)[51] | * | * | * | * | ** | * | * | * | 9 | High |
| Swiecki (2020)[55] | * | * | * | * | * | * | * | * | 8 | High |

| | | | | | | | | | | |
|---|---|---|---|---|---|---|---|---|---|---|
| Mosleh (2018)[53] | * | * | * | * | 0 | * | * | * | 7 | High |
